# Vapor–liquid–solid growth of large-area multilayer hexagonal boron nitride on dielectric substrates

**DOI:** 10.1038/s41467-020-14596-3

**Published:** 2020-02-12

**Authors:** Zhiyuan Shi, Xiujun Wang, Qingtian Li, Peng Yang, Guangyuan Lu, Ren Jiang, Huishan Wang, Chao Zhang, Chunxiao Cong, Zhi Liu, Tianru Wu, Haomin Wang, Qingkai Yu, Xiaoming Xie

**Affiliations:** 1 State Key Laboratory of Functional Materials for Informatics, Shanghai Institute of Microsystem and Information Technology, Chinese Academy of Sciences, 865 Changning Road, Shanghai, 200050 China; 2 School of Electronic, Electrical and Communication Engineering, University of Chinese Academy of Sciences, 19A Yuquan Road, Beijing, 100049 China; 3 CAS Center for Excellence in Superconducting Electronics (CENSE), 865 Changning Road, Shanghai, 200050 China; 4 State Key Laboratory of ASIC and System, School of Information Science and Technology, Fudan University, 220 Handan Road, Shanghai, 200433 China; 5 State Key Laboratory of Precision Spectroscopy, School of Physics and Material Science, East China Normal University, 3663N. Zhongshan Road, Shanghai, 200062 China; 6 School of Physical Science and Technology, ShanghaiTech University, 319 Yueyang Road, Shanghai, 200031 China

**Keywords:** Two-dimensional materials, Two-dimensional materials, Two-dimensional materials, Two-dimensional materials

## Abstract

Multilayer hexagonal boron nitride (*h*-BN) is highly desirable as a dielectric substrate for the fabrication of two-dimensional (2D) electronic and optoelectronic devices. However, the controllable synthesis of multilayer *h*-BN in large areas is still limited in terms of crystallinity, thickness and stacking order. Here, we report a vapor–liquid–solid growth (VLSG) method to achieve uniform multilayer *h*-BN by using a molten Fe_82_B_18_ alloy and N_2_ as reactants. Liquid Fe_82_B_18_ not only supplies boron but also continuously dissociates nitrogen atoms from the N_2_ vapor to support direct *h*-BN growth on a sapphire substrate; therefore, the VLSG method delivers high-quality *h*-BN multilayers with a controllable thickness. Further investigation of the phase evolution of the Fe-B-N system reveals that isothermal segregation dominates the growth of the *h*-BN. The approach herein demonstrates the feasibility for large-area fabrication of van der Waals 2D materials and heterostructures.

## Abstract (web summary)

Multilayer hexagonal boron nitride (hBN) is a desirable dielectric substrate for 2D electronics but its controllable synthesis is challenging. Here, the authors report a vapor–liquid–solid growth method to achieve uniform multilayer hBN by using a molten Fe_82_B_18_ alloy and N_2_ as reactants.

## Introduction

Recently, two-dimensional (2D) materials have received considerable attention as promising candidates for next-generation electronic and optoelectronic devices. However, they are very sensitive to their surroundings due to their 2D nature^1,2^. Hexagonal boron nitride (*h*-BN), a typical dielectric substrate, could maintain the intrinsic properties of other 2D materials due to its atomic flatness and the absence of dangling bonds and charge impurities on its surface^3–5^. Extensive research has shown the outstanding performance of multilayer *h*-BN as a substrate in electronic devices made from 2D materials^1,4^. Therefore, the fabrication of high-quality *h*-BN over a large area provides promise for its application in 2D electronics and optoelectronics^6–9^.

Through mechanical exfoliation^10^, high-quality multilayer *h*-BN flakes can be obtained from bulk *h*-BN crystals, which are typically synthesized by a metal-B-N solvent-assisted method under extreme temperatures (1500−1750 °C) and pressures (4.0−5.5 GPa)^11^. Limitations on the control of the lateral size and thickness prevent exfoliated *h*-BN from applications that require scaling. Therefore, much effort has been devoted to the mass production of large-area multilayer *h-*BN films. Currently, chemical vapor deposition (CVD) is a very successful method to grow *h*-BN on metals and alloys at relatively low cost^12–19^. However, catalytic effects always yield thin *h*-BN films, e.g., a thickness of a few layers (<10 layers), which could not efficiently protect electronic 2D crystals from the disturbance in their surroundings. The existing fabrication approaches use flammable and toxic chemicals, including borazine (B_3_N_3_H_6_)^16^, or complicated chemicals, such as ammonia borane (H_3_NBH_3_)^20^, as reactive precursors. In addition, the wet transfer process from metallic surfaces to target substrates would probably degrade the quality of the *h*-BN and then limit its application in microelectronics. We also noticed that there have been several reports about the direct synthesis of *h*-BN films on dielectric substrates^21,22^, but the low thickness control and high crystallinity of *h*-BN films are issues that remain elusive^21–24^.

**Fig. 1:**
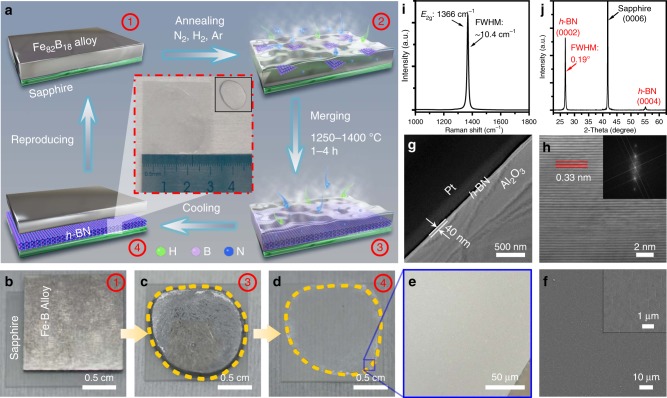
Fabrication of large-area multilayer *h*-BN on sapphire. **a** Schematics of multilayer *h*-BN grown on sapphire with Fe_82_B_18_ alloy and nitrogen as reactants. **b**–**d** OM images of Fe–B alloy on sapphire before and after the growth process. **e**, **f** OM and SEM images of multilayer *h*-BN on sapphire. **g** Cross-sectional TEM image of multilayer *h*-BN on sapphire. **h** High-resolution TEM image corresponding to (**g**). The fast Fourier transform (FFT) pattern is displayed in the inset. **i**, **j** Typical Raman and XRD spectra of multilayer *h*-BN film on sapphire.

In this study, we demonstrated a vapor–liquid–solid growth (VLSG) method to fabricate high-quality multilayer *h*-BN films on (0001) sapphire substrates using an Fe_82_B_18_ alloy and N_2_ as reactants. The melting point of the Fe_82_B_18_ (~1180 °C) is lower than that of most catalytic metal-B alloys. Besides, compared to commonly used NH_3_, N_2_ is a safer choice and would be friendly to environment. The VLSG, first proposed by Wagner in 1964, is widely used for one-dimensional crystal growth^25–28^. Here, we extended the method to the growth of 2D crystals. Figure 1a shows schematics of the VLSG method for multilayer *h*-BN growth. In the inset, our work demonstrated the reliable synthesis of large-area uniform multilayer *h*-BN on the surface of a 3 cm × 3 cm sapphire substrate. The current size of the *h*-BN films was due to the limited space and apparatus design of our CVD system only. The optical microscopy (OM) images were obtained at different stages and are presented in Fig. 1b–e (see detailed growth processes in the “Methods” section). We propose that the liquid catalyst (molten Fe_82_B_18_ alloy) promoted the dissociation of N_2_, served as a solvent for B–N associate diffusion through individual atomic vacancies and assisted the nucleation and 2D lateral growth of the solid *h*-BN film at the liquid–solid interface. This method has the potential to scale to wafer-sizes production of high-quality multilayer *h*-BN, which would boost practical applications of 2D materials and their van der Waals (vdW) hetero-structures.

## Results

### VLSG of thickness-controllable multilayer *h*-BN on sapphire

The investigation by scanning electron microscopy (SEM) verified the successful growth of a continuous and uniform *h*-BN film on sapphire substrate (Fig. 1f). The cross-sectional transmission electron microscopy (TEM) image shows a typical thickness of ~40 nm and an interlayer distance of 0.33 nm (Fig. 1g, h and Supplementary Fig. 1d). Furthermore, the TEM images of the *h*-BN film suspended on a TEM grid exhibit a smooth *h*-BN film without any ad-layers (Supplementary Fig. 1c, e and f). Moreover, as measured by ultraviolet–visible (UV–Vis) absorption spectroscopy, the intrinsic bandgap energy of the *h*-BN film was ~5.9 eV (Supplementary Fig. 1a, b), which is in agreement with previous reports^29,30^. The Raman spectrum of the multilayer *h-*BN transferred on SiO_2_ (300 nm)/Si substrate exhibits the characteristic peak at 1366 cm^−1^, and the full width at half maximum (FWHM) of this peak was measured to be 10.4 cm^−1^, which is comparable to those of the *h*-BN flakes exfoliated from the *h*-BN crystals (Fig. 1i). Additionally, the FWHM mapping of the E_2g_ mode displayed a high uniformity and low density of defects over a large area of the *h*-BN film (Supplementary Fig. 2). The X-ray diffraction (XRD) spectrum (Fig. 1j) displays the dominant (0006) peak of the sapphire ***c***-plane at 41.7°. In addition, the (0002) and (0004) *h*-BN peaks were observed at 26.6° and 55.2°, respectively, confirming that these multilayers were well aligned to the ***c*** axis that was perpendicular to the sapphire substrate^12^. The FWHM of the (0002) *h*-BN peak was measured to be 0.19°, indicating the highly crystalline quality of the *h*-BN multilayers^24^.

**Fig. 2:**
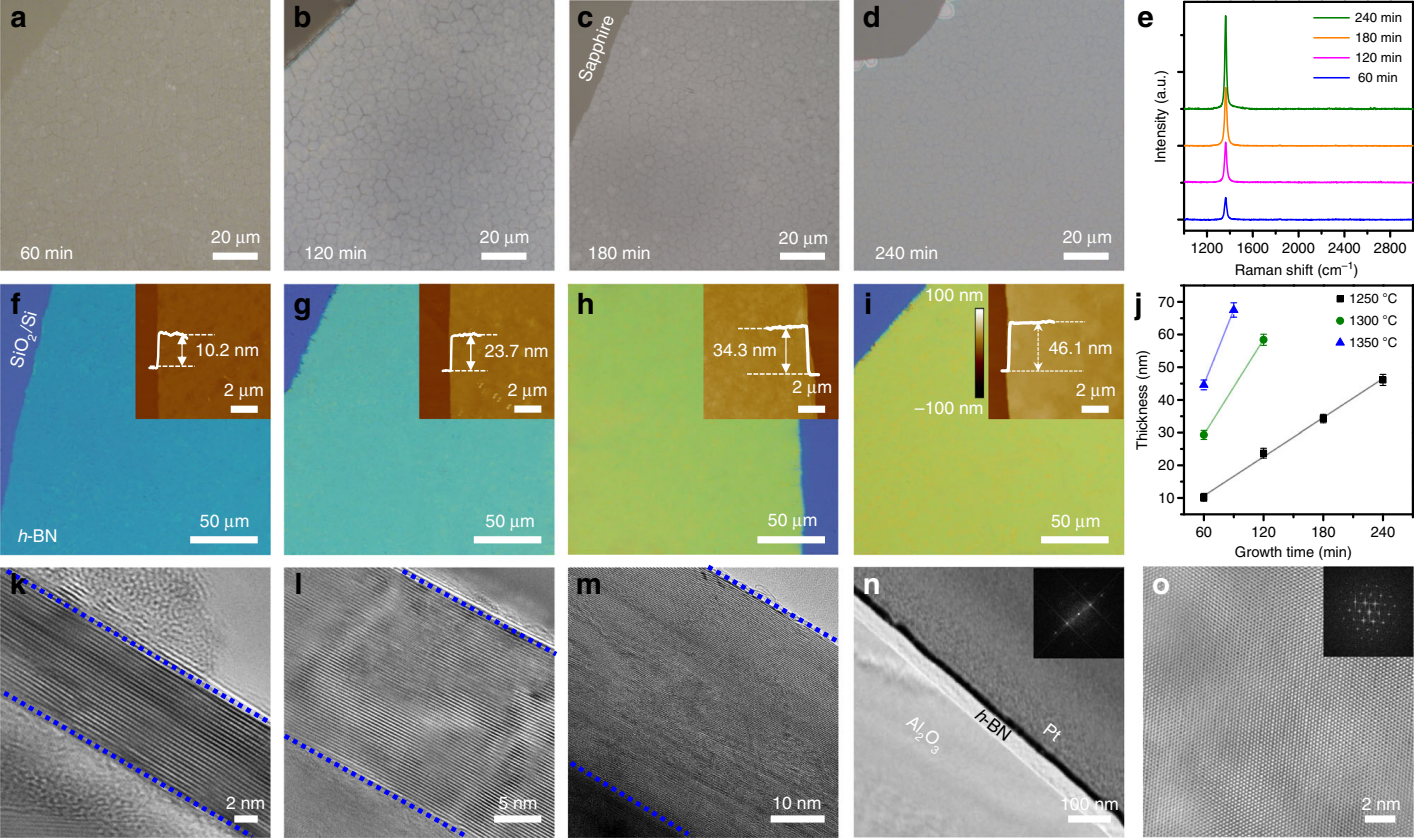
Controllable thickness of large-area multilayer *h*-BN on sapphire. **a**–**d**, Typical OM images of multilayer *h*-BN grown on sapphire with 60 (**a**), 120 (**b**), 180 (**c**) and 240 (**d**) minutes at 1250 °C. **e** Corresponding Raman spectra of multilayer *h*-BN with different growth times. **f**–**i** Corresponding to (**a**–**d**), multilayer *h*-BN are transferred onto SiO_2_ (300 nm)/Si substrate. The inset shows the corresponding AFM images. **j** Relationship between growth time and thickness of multilayer *h*-BN. Error bars for the experimental data represent standard deviation uncertainty of thicknesses collected from different samples. **k**–**n** Corresponding TEM images of multilayer *h*-BN with different thicknesses. **o** Typical TEM image and FFT pattern (inset) of multilayer *h*-BN.

During the VLSG method, the thickness of the *h*-BN can be simply controlled by adjusting the growth time and temperature. Figure 2a–d presents typical OM images of *h*-BN films grown on sapphire with different growth times from 60 to 240 min at 1250 °C. Figure 2f–i shows the *h*-BN films (corresponding to the films in Fig. 2a–d, respectively) that were transferred onto SiO_2_ (300 nm)/Si substrates. It was observed that the wrinkles caused by the mismatch of the thermal expansion coefficient between the sapphire and multilayer *h*-BN were completely released after the transfer. We systemically investigated the wrinkles, and the results are given in supplementary Figs. 3–5. The as-transferred *h*-BN multilayers exhibit a homogeneous color contrast, indicating a macroscopic uniformity in their thickness. Atomic force microscopy (AFM) was further used to determine the thickness and uniformity of the *h*-BN films (insets in Fig. 2f–i and supplementary Figs. 6–8). The thickness of the *h*-BN film linearly increased according to the growth time (Fig. 2j), and the *h*-BN film transferred on SiO_2_ (300 nm)/Si substrate had a very smooth surface over 10 μm × 10 μm area (root-mean-square roughness (*R*_a_) = 0.11 nm). The Raman spectra of the transferred *h*-BN showed a characteristic E_2g_ band at 1366~1368 cm^−1^ using a 532 nm laser excitation (Fig. 2e). The intensity of the E_2g_ band increased linearly with growth time after normalizing the intensity of characteristic silicon peak at 520 cm^−1^. The well-defined layer structure of the multilayer *h*-BN was analysed by means of high-resolution TEM (Fig. 2k–n). The orientation of the *h*-BN (0002) lattice plane was confirmed. In addition, as shown in Fig. 2o, the *h-*BN multilayer had highly ordered interlayer stacking. In the inset, the corresponding fast Fourier transform (FFT) pattern displays only one set of six-fold symmetric diffraction, indicating the well-defined stacking order of the as-prepared *h*-BN multilayers. Their stacking order was also verified via the method of H plasma treatment^31^. To control the thickness of the *h*-BN layers, the influence of the growth temperature was also investigated (Supplementary Fig. 9). The effect of the growth temperature/time on the thickness of multilayer *h-*BN is summarized in supplementary Table 1.

**Fig. 3:**
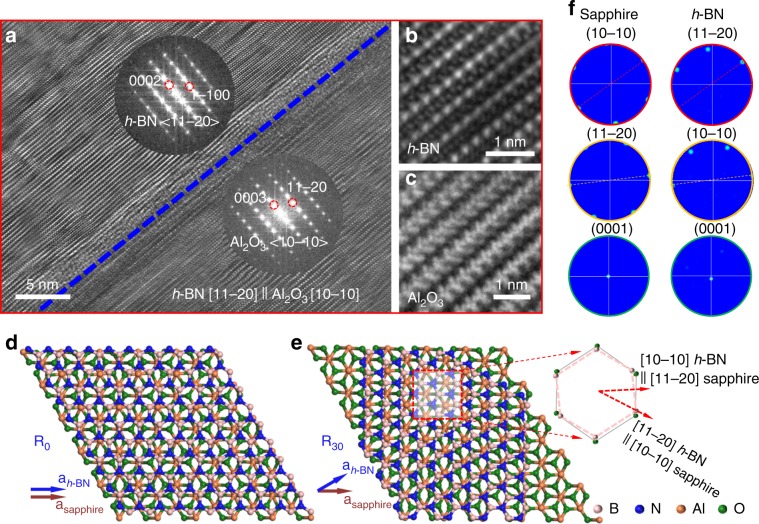
Epitaxial relationship between multilayer *h*-BN and sapphire. **a** High-resolution TEM image of <11-20> *h*-BN multilayers on sapphire along <10–10> of sapphire. The inset shows the corresponding FFT pattern from multilayer *h*-BN and sapphire areas, respectively. **b**, **c** TEM images with atomic resolution of multilayer *h*-BN (**b**) and sapphire (**c**). **d**, **e** Atomic arrangement of the *h*-BN layer on the sapphire substrate without rotation (R_0_) and with a rotational angle of 30° (R_30_). **f** EBSD pole figures in the (0001), (10–10), and (11–20) planes for *c*-plane sapphire substrate and multilayer *h*-BN.

The epitaxial relationship between the multilayer *h*-BN and sapphire was suggested by cross-sectional TEM and electron backscattered diffraction (EBSD). A cross-sectional TEM image displays the interface between *h*-BN and sapphire (Fig. 3a). According to the corresponding FFT patterns of the as-grown *h*-BN and sapphire (Fig. 3a insets), it was calculated that the [11-20] direction in the *h*-BN was well aligned to the [10-10] direction in the sapphire. In addition, the atomic resolution and atomic fringes in the magnified high-resolution TEM images of *h*-BN and sapphire also confirmed the alignment (Fig. 3b–e). Furthermore, as shown in Fig. 3f, the pole figures of the sapphire and *h*-BN films proved that the alignment of the *h*-BN layers on sapphire was [0001] *h*-BN||[0001] sapphire, [10-10] *h*-BN||[11-20] sapphire, and [11-20] *h*-BN||[10-10] sapphire, consistent with previous reports^21,29^.

### Mechanism of VLSG of multilayer *h*-BN on Fe_82_B_18_ alloy

**Fig. 4:**
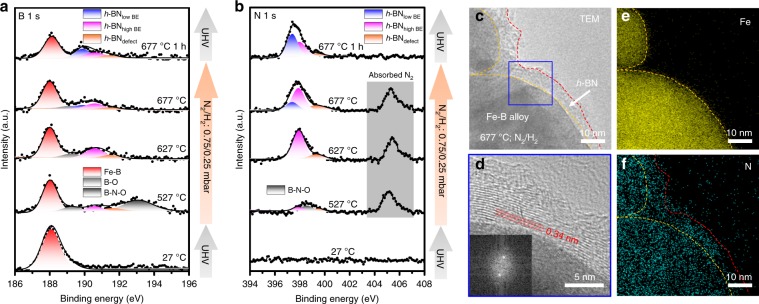
Investigation of the *h*-BN growth process on Fe-B alloy. **a**, **b** B 1s (**a**) and N 1s (**b**) spectra during the in situ APXPS measurement. **c**, **d** TEM images of Fe–B nanoparticles wrapped with layered *h*-BN. **e**, **f** EDS mapping of Fe (**e**) and N (**f**) corresponding to (**c**).

To explore how B-N bonding initially forms in Fe-B alloy, we carried out in situ ambient pressure X-ray photoelectron spectroscopy (APXPS) measurements on the surface of the Fe-B alloy. The temperature-resolved evolution of the B 1s and N 1s spectra were investigated from 27 °C (300 K) to 677 °C (950 K) in a N_2_/H_2_ atmosphere, which indicated the decomposition of N_2_ and formation of the B-N phase on the Fe-B alloy during the heating process (Fig. 4a, b). Here, high binding energy (BE) and low BE peak pairs, depicted as the characteristic peak of monolayer and few-layer *h*-BN, respectively, are centered at 190.6/397.9 eV and 189.9/397.5 eV, respectively, consistent with a previous report^14^. In addition, the B/N peak pair located at 191.4/399.4 eV was identified as a defect species^32^. With increasing temperature, the increase in the intensity ratio of the lower BE and higher BE B/N peak pairs indicates that few-layer *h*-BN formed gradually instead of a monolayer^14^. After annealing, the B/N atomic ratio was approximately 1, which is consistent with the multilayer *h*-BN synthesized at 1250 °C (Supplementary Fig. 10a–f). Moreover, ex situ TEM and energy dispersive spectrometry (EDS) mapping of Fe and N demonstrated that layered *h*-BN formed at 677 °C on Fe-B nanoparticles by annealing in a N_2_ /H_2_ atmosphere for 6 h (Fig. 4c–f).

Previous studies^14–16^ reported that N was almost completely insoluble in Cu, Ni, and Fe at 1000 °C. In this work, it was found that the Fe-B alloy played a critical role in both the decomposition of N_2_ and the formation of few-layer *h*-BN via in situ APXPS experiments. The utilization of the Fe-B alloy broke the surface-limited growth process, which always yielded monolayer *h*-BN. Moreover, under a high temperature of 1250 °C, the efficiency of both the decomposition of N_2_ and formation of B-N associates was improved, and then the growth rate of the multilayer *h*-BN increased. Time-resolved N 1s evolution at 577 °C (850 K) in a N_2_/H_2_ atmosphere and growth experiments at 1250 °C for 120 min with different cooling rates were carried out the confirm the isothermal process (Supplementary Figs. 10g, h and 11). We propose that the abundant B in the alloy activated the isothermal segregation of the *h*-BN layers. In contrast to the previous growth mechanism for *h*-BN, the isothermal growth procedure on an active melted Fe–B alloy greatly improved the uniformity, crystallinity and smoothed surface of the *h*-BN layers. It may suppress the formation of thick *h*-BN flakes induced by cooling-induced segregation.

According to the heterogeneous equilibrium in the B–Fe–N phase diagram, the N and B in the liquid comprise Fe, BN, Fe_2_B, and atomic nitrogen (details in Supplementary Figs. 12 and 13)^33^. The growth process at high temperatures can be divided into three stages as follows. First, the Fe-B alloy melted at 1250 °C in a N_2_ atmosphere. As a precursor during this stage, the N_2_ decomposed at the vapor–liquid interface and reacted with B to form chemically stable B–N associates, which was illustrated through the in situ APXPS experiments. Second, the liquid phase broke the stability of the lattice in the solid alloy and then produced enough vacancies in the lattice, which increased the diffusion of the B–N associates^34^. Crystalline *h*-BN structures formed at the liquid–solid interface between the molten alloy and sapphire. Finally, the B–N associates continuously diffused through the liquid and interacted with the Fe catalyst. The formation of new *h*-BN layers between the preformed *h*-BN and Fe–B alloy ensured the controllable synthesis of high-quality multilayer *h*-BN. The synthesis of *h*-BN here relied on the formation and diffusion of B–N associates during the reaction.

### Applications of CVD multilayer *h*-BN

The multilayer *h*-BN transferred onto a SiO_2_ (300 nm)/Si substrate was investigated by room-temperature Raman and photoluminescence (PL) spectroscopies for determining the optically active defect distribution^35–41^. As shown in supplementary Fig. 14a and b, Raman mapping of the E_2g_ intensity with the corresponding OM and AFM images demonstrated the uniformity of the fabricated *h*-BN layers. The subsequent PL intensity mapping demonstrated that optically active defects were only present at edges and wrinkles. The PL spectra at those typical spots were extracted and fitted by Lorentzian fitting (supplementary Fig. 14c). On one hand, three peaks centered at 2.13, 1.97, and 1.81 eV are observed in the wrinkled areas, which is consistent with zero phonon line (ZPL) and corresponding one- and two-optical-phonon sidebands (PSB)^37,39^. These optical emissions show coincident features with a previous report, which claimed that wrinkle-induced emissions are sensitive to strain and/or local lattice symmetry distortions^39^. On the other hand, the edges of a *h*-BN flake on the SiO_2_ (300 nm)/Si substrate show a different optical emission. The ZPL peak is located at 2.06 eV, and the corresponding PSB are evident at 1.90 and 1.74 eV. Moreover, the energy difference between the two adjacent peaks in all optical emissions is ~160 meV, which agrees well with the in-plane optical (LO/TO) phonon energy of *h*-BN^42^. Additionally, an overlapping multilayer *h*-BN on a SiO_2_ (300 nm)/Si substrate was studied by Raman and PL, showing similar results (supplementary Fig. 14d–f). The fact that no optical emissions at energies between 1.7 and 2.2 eV could be found in large uniform areas provides the possibility to artificially fabricate optically active defects for optoelectronics in the future^40^.

**Fig. 5:**
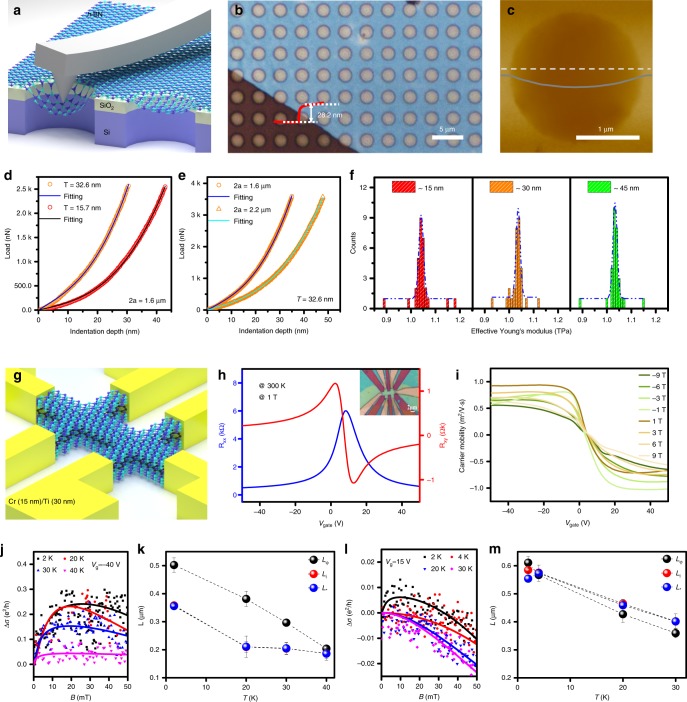
Mechanical strength of multilayer *h*-BN film and electron transport of the *h*-BN/graphene/*h*-BN device. **a** Schematic of nanoindentation on suspended multilayer *h*-BN film. **b** OM image of *h*-BN membranes lying on SiO_2_ (300 nm)/Si substrate with patterned circular wells. **c** AFM image of one membrane, 2.2 μm in diameter. The solid line is a height profile along the dashed line. **d** Measured force-displacement curves of multilayer *h*-BN with different thicknesses. **e** Measured force-displacement curves of multilayer *h*-BN with different film diameters. **f** Histogram of Young’s modulus with different thicknesses. Dashed lines represent Gaussian fits to data. **g** Schematic diagram of the *h*-BN/graphene/*h*-BN Hall bar configuration. **h** The relationship of *R*_*xx*_ and *R*_*xy*_ versus gate voltage (*V*_g_) at 300 K under 1 T. An OM image of the *h*-BN/graphene/*h*-BN Hall bar device is shown in the inset. **i** Magnetic field dependence of the carrier mobility versus *V*_g_. **j** Normalized magneto-conductivity (MC) measured at different temperatures with *V*_g_ at approximately −40 V; those solid lines are fitting curves. **k** Temperature dependence of three characteristic lengths (L_φ,_ L_i,_ L_∗_) extracted from (**j**). **l** Normalized MC measured at low temperatures with *V*_g_ maintained at 15 V. **m** Temperature dependence of L_φ,_ L_i_ and L_∗_ extracted from (**l**). Error bars in (**k**) and (**m**) for the experimental data represent standard deviation of uncertainty in characteristic lengths (L_φ_, L_i,_ L_∗)_ extraction.

The mechanical strength of the multilayer *h*-BN was investigated with AFM nanoindentation (Fig. 5a). First, the *h*-BN films were transferred onto a SiO_2_ (300 nm)/Si substrate patterned with circular holes. As shown in Fig. 5b, the OM image of the multilayer *h*-BN suspended on an array of circular holes indicates high uniformity. Subsequent AFM measurements indicated that the *h*-BN films were stretched tautly across the hole openings (Fig. 5c). By indenting the free-standing film at the center of the holes, the mechanical deformation of the *h*-BN film was explored. Figures 5d and e present examples of the force-displacement behavior of two multilayer *h*-BN flakes with thicknesses of 15.7 and 32.6 nm that were lying on circular holes with diameters of 1.6 and 2.2 μm, respectively. The Young’s modulus of the multilayer *h*-BN flakes was derived by fitting the force-displacement curves^16,43^. We measured 30 positions for each piece of *h*-BN flake in the uniform thickness, and the distribution of the derived values was plotted in Fig. 5f. The values of Young’s modulus were approximately 1.04 ± 0.1 TPa, which agrees within experimental error with the theoretical value^12,16^. The results indicated that the high mechanical strength of these multilayer *h*-BN films was independent of their thickness.

To evaluate the applicability of the multilayer *h*-BN as a dielectric, graphene sandwiched between multilayer *h-*BN films was patterned into a Hall bar configuration, and the electronic performance of graphene was evaluated (Fig. 5g). The longitudinal resistance (*R*_*xx*_) and transversal resistance (*R*_*xy*_) of the device were measured under 1 T at 27 ^°^C (300 K), and the OM image of the device is shown in the inset (Fig. 5h). The carrier mobility of graphene at 27 ^°^C (300 K) under different magnetic fields, extracted by *μ* = 1/*B*∙(*R*_*xy*_∙*L*)/ (*R*_xx_∙*W*)^1^, was in the range of 0.5~1 m^2^ V^−1^ s^−1^ (Fig. 5i). As a baseline, the carrier mobility of CVD-grown graphene transferred onto SiO_2_ (300 nm)/Si substrate was just slightly higher than 0.3 m^2^ V^−1^ s^−1^ (Supplementary Fig. 16). Direct growth of graphene on *h*-BN^44^ or cleaner transfer of graphene^45^ could achieve better performance of field effect devices. Furthermore, the magneto-conductivity (MC) was measured at low temperatures with different back-gate biases (*V*_g_) to investigate the weak localization (WL) and weak anti-localization (WAL) phenomenon of graphene (Fig. 5j–m). For this study, we used the expression for the WL-induced conductivity correction as theoretically suggested with three parameters: the phase coherence length (*L*_φ_), elastic intervalley scattering length (*L*_i_) and intravalley scattering length (L_*_)^46^. When *V*_g_ was at −40 V, a positive MC was collected, which clearly presented the WL effect (Fig. 5j). Both intravalley and intervalley scattering were strong, such that *L*_φ_ ≫ *L*_i_ ≳ L_*_ (Fig. 5k). However, when *V*_g_ was fixed at approximately 15 V, the WL was transformed into WAL with increasing temperature (Fig. 5l). In addition, *L*φ < *L*_i_, L_*_ (Fig. 5m). Overall, the distance of each of two defects in the *h*-BN was larger than the length of the scattering (approximately 400 nm), which was comparable to that in a previous report^47^.

## Discussion

In summary, high-quality multilayer *h-*BN with a controllable thickness was achieved on sapphire via the VLSG method. The use of molten Fe_82_B_18_ enabled the uniform isothermal segregation of multilayer *h*-BN with a thickness of 5~50 nm. The 2D growth was strictly limited to the interface between the liquid Fe_82_B_18_ and the sapphire substrate. The in situ APXPS helped us to observe how the B-N associates initially formed and understand the associated segregation mechanism. The existence of vacancies in the liquid alloy increased the diffusion of the B-N associates and then accumulated the multilayer *h*-BN formed at high temperature. Moreover, the PL, Raman, AFM nano-indentation and electron transport measurements provided evidence for the fabrication of high-quality multilayer *h-*BN. The VLSG method demonstrated an efficient method for the synthesis of *h*-BN. The approach exhibited potential for large-area synthesis of multilayer *h*-BN and integration of other 2D materials.

## Methods

### Fabrication of Fe_82_B_18_ alloy

Following the Fe-B binary phase diagram, Fe_2_B and Fe were chosen as initial reactants, and the molar ratio between Fe_2_B and Fe was set as 1:2.3. The raw materials were mixed thoroughly and annealed at 1500 °C for 120 min. Then, the samples were cooled naturally. The whole process was maintained at ambient pressure under Ar flows.

### Synthesis of multilayer *h-*BN on sapphire

Multilayer *h-*BN was synthesized on sapphire with a (0001) facet under ambient pressure by using an Fe_82_B_18_ alloy and N_2_ as reactants. First, a plate of Fe-B alloy was placed on top of the sapphire and loaded into the reaction chamber. The system was annealed at 1,250 °C for 60 min under a mixed Ar/H_2_ (300/50 sccm) ambient atmosphere. Then, N_2_ was introduced with a flux of 300 sccm at 1250 °C for 60 min. The *h*-BN multilayer isothermally segregated on the interface between the sapphire and Fe-B alloy. Finally, the reaction chamber cooled to room temperature at a rate of 10 °C/min under a mixed Ar/H_2_ (300/50 sccm) gas. The Fe–B alloy could be easily peeled off and left the *h*-BN multilayer on the sapphire substrate.

### *h*-BN Characterizations

Surface morphology and crystalline properties of the *h*-BN layers were investigated by SEM (Zeiss supra55, operated at 1.5 kV), AFM (Bruker Dimension Icon, tapping mode) and XRD (Bruker D8 DISCOVER). TEM (JEM-2100F, operated at 200 kV), PL & Raman spectroscopy (WITec Alpha 300 R, 532 nm laser with ×50 objective lens, operated at room temperature) and UV–Vis absorption spectroscopy (Agilent EV 300) evaluates the quality of as-synthesized multilayer *h*-BN preliminarily. In situ XRD (Bruker D8 ADVANCE) and APXPS (Species, Al Kα) were used to understand the phase transition in Fe-B alloy and the growth mechanism of multilayer *h*-BN. TEM (JEM-ARM 300 F, operated at 80 kV), focused ion beam (FIB, Helios Nanolab 600) and EBSD (Bruker *e*^−^Flash^FS^) were employed to understand the epitaxial relationship between multilayer *h*-BN and sapphire substrate. For mechanical characterizations, a diamond-coated AFM tips (Tap190DLC, Budget sensors) was used and the spring constant of the AFM cantilever was calibrated as 75.4 N m^−1^.

### In situ APXPS and *XRD* characterization

For in situ APXPS measurements, the Fe_82_B_18_ alloy was cleaned by Ar+ sputtering to remove surface residues. During the heating process, a N_2_/H_2_ (3:1) mixed gas was introduced into the chamber, and the pressure was kept at 1 mbar constantly. When the temperature reached the set point, the N 1s spectrum and B 1s spectrum were collected. Finally, the Fe_82_B_18_ alloy was cooled to room temperature naturally. For in situ XRD measurements, the XRD spectra of the original Fe–B alloy were measured in advance for further comparison. First, N_2_ (400 sccm) was introduced into the chamber at ambient atmosphere and flowed during the whole process. Then, the stage began to heat at a heating rate of 10 °C min^−1^. When the temperature reached the set point, the measurement process consumed ~30 min for each calibration. After that, the system was cooled down at a rate of 10 °C min^−1^. Two additional temperature spots were calibrated during the cooling process. Finally, the Fe–B alloy was cooled to room temperature naturally.

### Electronic device fabrication

The *h*-BN/graphene/*h*-BN van der Waals (vdW) hetero-structure was assembled by the “peel and stack” technique. To fabricate the hetero-structure, we started with as-transferred monolayer graphene on top of an oxidized Si wafer. The monolayer graphene was synthesized via CVD method and the growth process had been reported in earlier literature^48^. The graphene monolayer was picked up by *h*-BN multilayer attached to a PDMS/PPC membrane and then stacked on to another freshly cleaved *h*-BN film that was grown. The detailed procedures illustrated graphically in Supplementary Fig. 15. The “peel and stack” technique ensured a clean interface between the flakes. Then, the hetero-structure was shaped into a Hall bar with a channel length of 4 μm and a width of 2 μm. After that, magnetron sputtered Cr (15 nm)/Ti (30 nm) defined multiple electrodes for transport measurement.

## Supplementary information

Supplementary Information

## Data Availability

All data is available in the main text or the supplementary materials.

## References

[1] Dean CR (2010). Boron nitride substrates for high-quality graphene electronics. Nat. Nanotechnol..

[2] Chen J-H, Jang C, Xiao S, Ishigami M, Fuhrer MS (2008). Intrinsic and extrinsic performance limits of graphene devices on SiO_2_. Nat. Nanotechnol..

[3] Wang L (2013). One-dimensional electrical contact to a two-dimensional material. Science.

[4] Bediako DK (2018). Heterointerface effects in the electrointercalation of van der Waals heterostructures. Nature.

[5] Li L (2016). Quantum Hall effect in black phosphorus two-dimensional electron system. Nat. Nanotechnol..

[6] Geim AK, Grigorieva IV (2013). Van der Waals heterostructures. Nature.

[7] Ni GX (2015). Plasmons in graphene moiré superlattices. Nat. Mater..

[8] Chen L (2017). Oriented graphene nanoribbons embedded in hexagonal boron nitride trenches. Nat. Commun..

[9] Lu G (2017). Synthesis of high-quality graphene and hexagonal boron nitride monolayer in-plane heterostructure on Cu-Ni alloy. Adv. Sci..

[10] Huang Y (2015). Reliable exfoliation of large-area high-quality flakes of graphene and other two-dimensional materials. ACS Nano.

[11] Watanabe K, Taniguchi T, Kanda H (2004). Direct-bandgap properties and evidence for ultraviolet lasing of hexagonal boron nitride single crystal. Nat. Mater..

[12] Kim SM (2015). Synthesis of large-area multilayer hexagonal boron nitride for high material performance. Nat. Commun..

[13] Lu G (2015). Synthesis of large single-crystal hexagonal boron nitride grains on Cu–Ni alloy. Nat. Commun..

[14] Caneva S (2016). Controlling catalyst bulk reservoir effects for monolayer hexagonal boron nitride CVD. Nano Lett..

[15] Shi Y (2010). Synthesis of few-layer hexagonal boron nitride thin film by chemical vapor deposition. Nano Lett..

[16] Song L (2010). Large scale growth and characterization of atomic hexagonal boron nitride layers. Nano Lett..

[17] Kim KK (2012). Synthesis of monolayer hexagonal boron nitride on Cu foil using chemical vapor deposition. Nano Lett..

[18] Uchida Y (2018). Controlled growth of large-area uniform multilayer hexagonal boron nitride as an effective 2D substrate. ACS Nano.

[19] Lee JS (2018). Wafer-scale single-crystal hexagonal boron nitride film via self-collimated grain formation. Science.

[20] Babenko V (2017). Time dependent decomposition of ammonia borane for the controlled production of 2D hexagonal boron nitride. Sci. Rep..

[21] Jang A-R (2016). Wafer-scale and wrinkle-free epitaxial growth of single-orientated multilayer hexagonal boron nitride on sapphire. Nano Lett..

[22] Li Q (2018). Direct growth of 5 in. uniform hexagonal boron nitride on glass for high-performance deep-ultraviolet light-emitting diodes. Adv. Mater. Interfaces.

[23] Tay RY (2015). Direct growth of nanocrystalline hexagonal boron nitride films on dielectric substrates. Appl. Phys. Lett..

[24] Li X (2016). Large-area two-dimensional layered hexagonal boron nitride grown on sapphire by metalorganic vapor phase epitaxy. Cryst. Growth Des..

[25] Wagner RS, Ellis WC (1964). Vapor‐liquid‐solid mechanism of single crystal growth. Appl. Phys. Lett..

[26] Arenal R, Stephan O, Cochon J-L, Loiseau A (2007). Root-growth mechanism for single-walled boron nitride nanotubes in laser vaporization technique. J. Am. Chem. Soc..

[27] Morales AM, Lieber CM (1998). A laser ablation method for the synthesis of crystalline semiconductor nanowires. Science.

[28] Li S (2018). Vapour–liquid–solid growth of monolayer MoS_2_ nanoribbons. Nat. Mater..

[29] Kobayashi Y, Tsai C-L, Akasaka T (2010). Optical band gap of h-BN epitaxial film grown on *c*-plane sapphire substrate. Phys. Status Solidi.

[30] Kumbhakar P (2015). Nonlinear optical properties and temperature-dependent UV-Vis absorption and photoluminescence emission in 2D hexagonal boron nitride nanosheets. Adv. Opt. Mater..

[31] He L (2019). Isolating hydrogen in hexagonal boron nitride bubbles by a plasma treatment. Nat. Commun..

[32] Kidambi PR (2014). In situ observations during chemical vapor deposition of hexagonal boron nitride on polycrystalline copper. Chem. Mater..

[33] Tomashik, V. *Iron Systems, Part 1*. **11D1**, (Springer Berlin Heidelberg, 2008).

[34] Park S (2015). Formation of hexagonal boron nitride by metal atomic vacancy-assisted B–N molecular diffusion. ACS Nano.

[35] Grosso, G. et al. Tunable and high-purity room temperature single-photon emission from atomic defects in hexagonal boron nitride. *Nat. Commun*. **8**, (2017). 10.1038/s41467-017-00810-2 PMC5615041 28951591

[36] Tran TT, Bray K, Ford MJ, Toth M, Aharonovich I (2016). Quantum emission from hexagonal boron nitride monolayers. Nat. Nanotechnol..

[37] Jungwirth NR, Fuchs GD (2017). Optical absorption and emission mechanisms of single defects in hexagonal boron nitride. Phys. Rev. Lett..

[38] Schué L, Stenger I, Fossard F, Loiseau A, Barjon J (2016). Characterization methods dedicated to nanometer-thick hBN layers. 2D Mater..

[39] Li X (2017). Nonmagnetic quantum emitters in boron nitride with ultranarrow and sideband-free emission spectra. ACS Nano.

[40] Comtet J (2019). Wide-field spectral super-resolution mapping of optically active defects in hexagonal boron nitride. Nano Lett..

[41] Lončarić I (2018). Imaging of optically active defects with nanometer resolution. Nano Lett..

[42] Serrano J (2007). Vibrational properties of hexagonal boron nitride: inelastic X-ray scattering and ab initio calculations. Phys. Rev. Lett..

[43] Lee C, Wei X, Kysar JW, Hone J (2008). Measurement of the elastic properties and intrinsic strength of monolayer graphene. Science.

[44] Tang S (2015). Silane-catalysed fast growth of large single-crystalline graphene on hexagonal boron nitride. Nat. Commun..

[45] Banszerus L (2015). Ultrahigh-mobility graphene devices from chemical vapor deposition on reusable copper. Sci. Adv..

[46] Yang XC (2013). Magnetotransport of polycrystalline graphene: Shubnikov-de Haas oscillation and weak localization study. Appl. Phys. Lett..

[47] Tikhonenko FV, Horsell DW, Gorbachev RV, Savchenko AK (2008). Weak localization in graphene flakes. Phys. Rev. Lett..

[48] Wu T (2016). Fast growth of inch-sized single-crystalline graphene from a controlled single nucleus on Cu–Ni alloys. Nat. Mater..

