# Supplementary material for: Vapor–liquid–solid growth of large-area multilayer hexagonal boron nitride on dielectric substrates
Source: Nat Commun. 2020 Feb 12;11:849. doi: 10.1038/s41467-020-14596-3 (PMC7015929; doi:10.1038/s41467-020-14596-3)
Supplement: Supplementary file 1 — Supplementary Information [file 41467_2020_14596_MOESM1_ESM.pdf]

Supplementary Information for

# Vapor–liquid–solid Growth of Large Area Multilayer Hexagonal Boron Nitride on Dielectric Substrates

Zhiyuan Shi, et al.

## Characterizations of crystalline structure and intrinsic optical bandgap of multilayer *h*-BN

Intrinsic bandgap property of *h*-BN film was measured by UV-Vis absorption spectroscopy. A 600 ×450 $\mu m^2$ region, shown in supplementary Fig. 1a, indicates the uniformity of the *h*-BN film. The optical absorption spectrum and corresponding Tauc plot ($(\alpha h\upsilon)^2$ versus $h\upsilon$) reveal that the optical bandgap energy (OBE) is ~5.9 eV, which is consistent with previous reports (supplementary Fig. 1b) [1,2].

Supplementary Fig. 1c presents a low-resolution TEM image of synthesized *h*-BN film transferred onto a TEM grid. Cross-sectional TEM investigation proved that the thickness of the *h*-BN film is ~ 40 nm (supplementary Fig. 1d). The atomic structure of the sample is proved by the high-resolution TEM image (supplementary Fig. 1e). Corresponding fast Fourier transform (FFT) image displays clear lattice fringe, indicating high crystalline with hexagonal symmetry. Further, atomic resolution TEM image of multilayer *h*-BN shows the arrangement of boron and nitrogen atoms clearly (supplementary Fig. 1f).

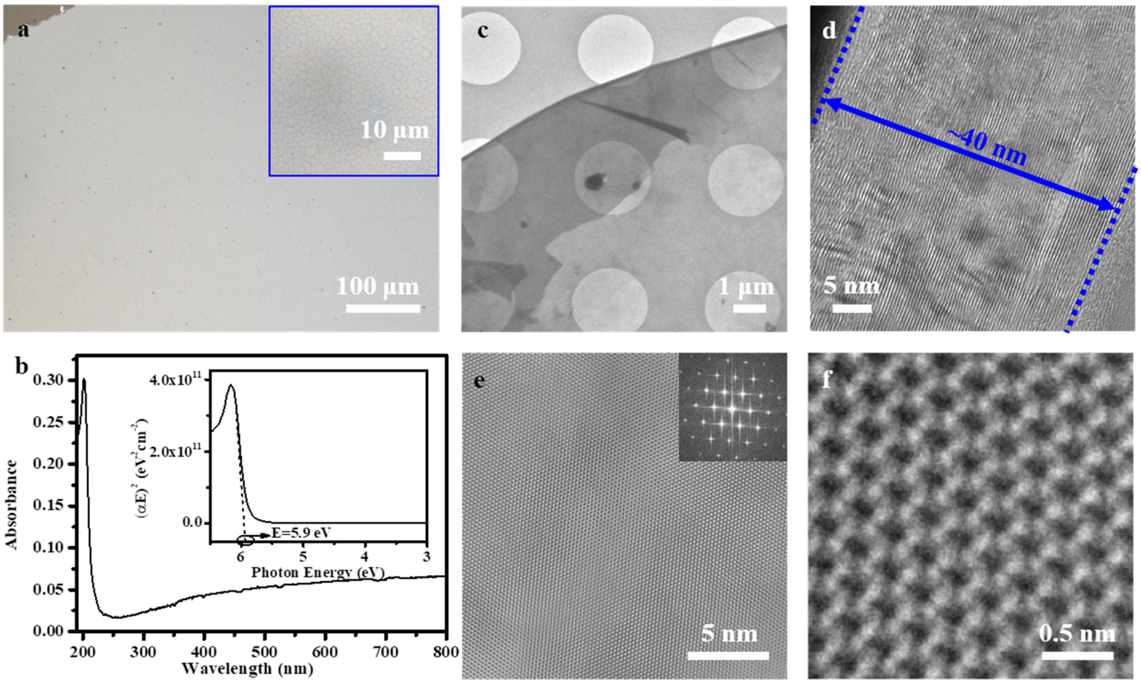


**Supplementary Figure 1 | Atomic structure and intrinsic optical bandgap of multilayer *h*-BN. a,** OM image of large area multilayer *h*-BN on sapphire. **b,** Optical absorption spectrum of typical multilayer *h*-BN. Inset shows Tauc plot, in which the OBE of multilayer *h*-BN is around 5.9 eV. **c,** Low-magnification TEM images of a *h*-BN film. **d,** A cross-sectional TEM image of a *h*-BN multilayer. **e and f,** High-magnification (**e**) and atomic resolution (**f**) TEM image of multilayer *h*-BN.

**Investigation on imperfections in a multilayered h-BN on sapphire**

Raman spectroscopy is an effective tool to characterize the imperfection in two-dimensional materials. As shown in Supplementary Fig. 2, large area multilayer *h*-BN was grown directly on sapphire substrate. We map the imperfection distribution in a location selected randomly by using Raman spectroscopy. The results are shown in Supplementary Fig. 2b and 2c. As shown in Supplementary Fig. 2b, the FWHM mapping of $E_{2g}$ mode exhibits a high homogeneity in contrast. It indicates a relatively low defect density.

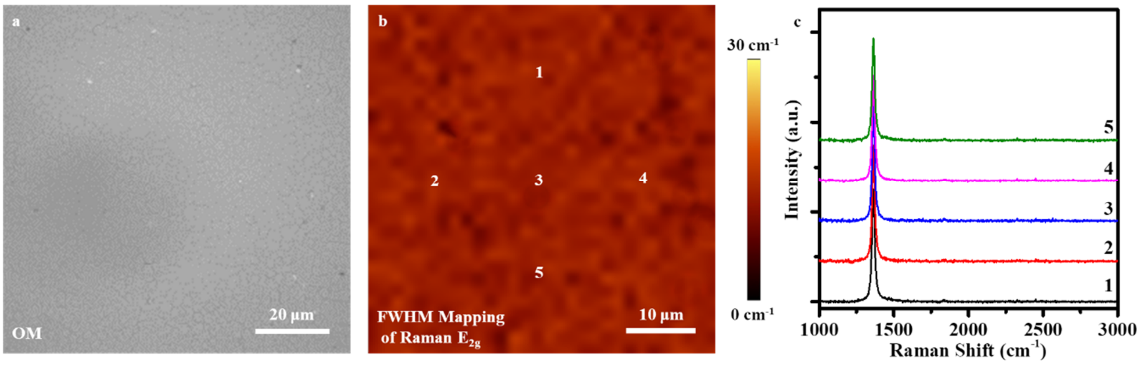


Supplementary Figure 2 | **Investigation in the imperfections on the as-grown multilayer *h*-BN. a**, OM image of large area *h*-BN thin film on sapphire. **b**, FWHM mapping of $E_{2g}$ mode of multilayer *h*-BN. **c**, Raman spectra randomly taken from five different positions on the multilayered *h*-BN.

## Characterization of *h*-BN wrinkles and bubbles

SEM, AFM and TEM measurements were carried out to investigate the origin of *h*-BN wrinkles on sapphire substrate. As shown in supplementary Fig. 3a, most of the wrinkle junctions (>95%) are 3-fold and form oriented honeycomb patterns, which shows similar features as mechanical exfoliated *h*-BN flakes after annealing at ~1250 °C (supplementary Fig. 3c). It proves the highly homogeneity of as-synthesized multilayer *h*-BN indirectly. Further, *h*-BN films transferred onto $SiO_2$ (300 nm)/Si substrate present smooth and uniform surface. It indicates that the thermal strain induced wrinkles could be effectively released (supplementary Fig. 3b). The structure features in the flat area and in the localized wrinkle have been studied using cross-section TEM. Figure S3D displays hollow structures under the wrinkles on the substrate. The lattice bending due to the compressive strain could clearly be observed. The stacks of as-grown basal planes are still highly oriented, but they are bent following the wrinkling waviness. The flat area of *h*-BN layer shows a highly ordered layered lattice, which proves the well-defined stacking sequence of as-grown multilayer *h*-BN (supplementary Fig. 3e).

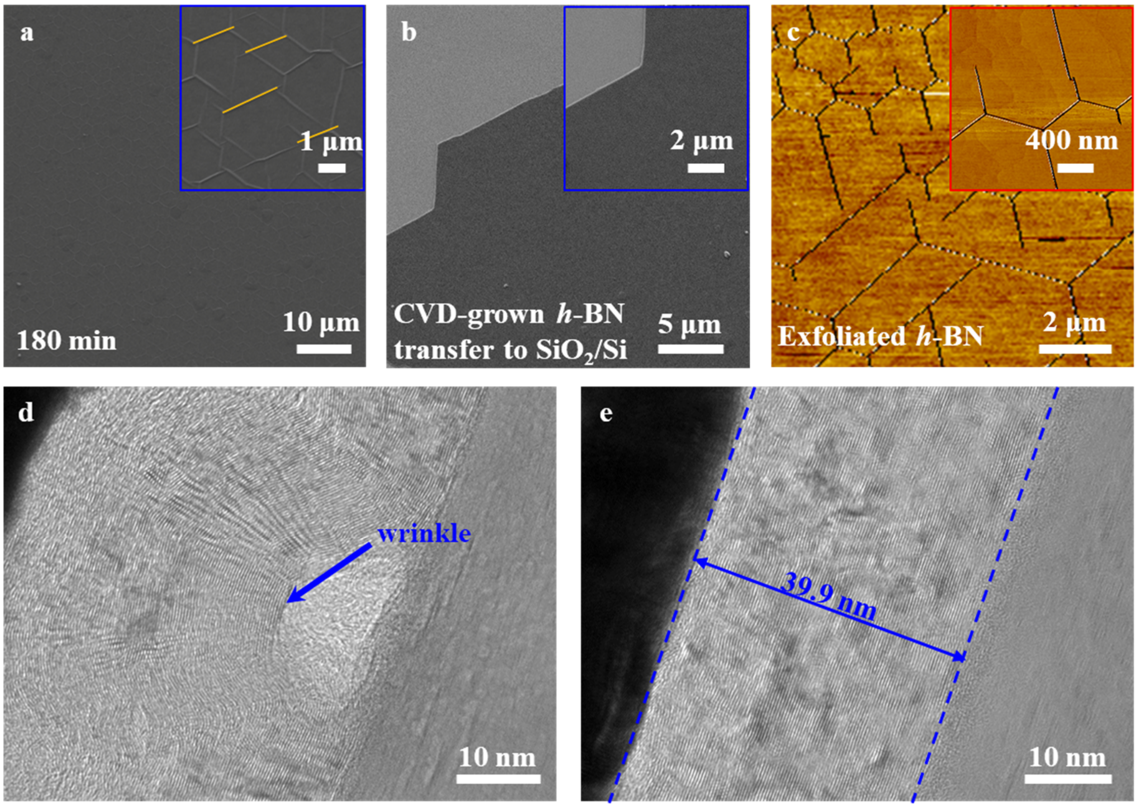


**Supplementary Figure 3 | Characterization of *h*-BN wrinkles. a,** SEM image of typical *h*-BN wrinkles formed on sapphire substrate. The inset shows enlarged SEM image. **b,** CVD-grown *h*-BN was transferred onto $SiO_2$ (300 nm)/Si substrate, which shows smooth surface. The inset shows enlarged SEM image. **c,** AFM image of exfoliated *h*-BN on sapphire after annealing. **d and e,** Corresponding cross-sectional TEM images of multilayer *h*-BN.

Sometimes, a few multilayer *h*-BN bubbles with clear newton rings were formed randomly on sapphire (supplementary Fig. 4a). The bubbles should be caused by the weak vdW interactions between multilayer *h*-BN and substrate due to the contamination of sapphire before the growth procedure. Corresponding SEM image in supplementary Fig. 4b shows smooth and uniform morphology of suspended *h*-BN bubbles, which release the thermal strain energy generated during cooling.

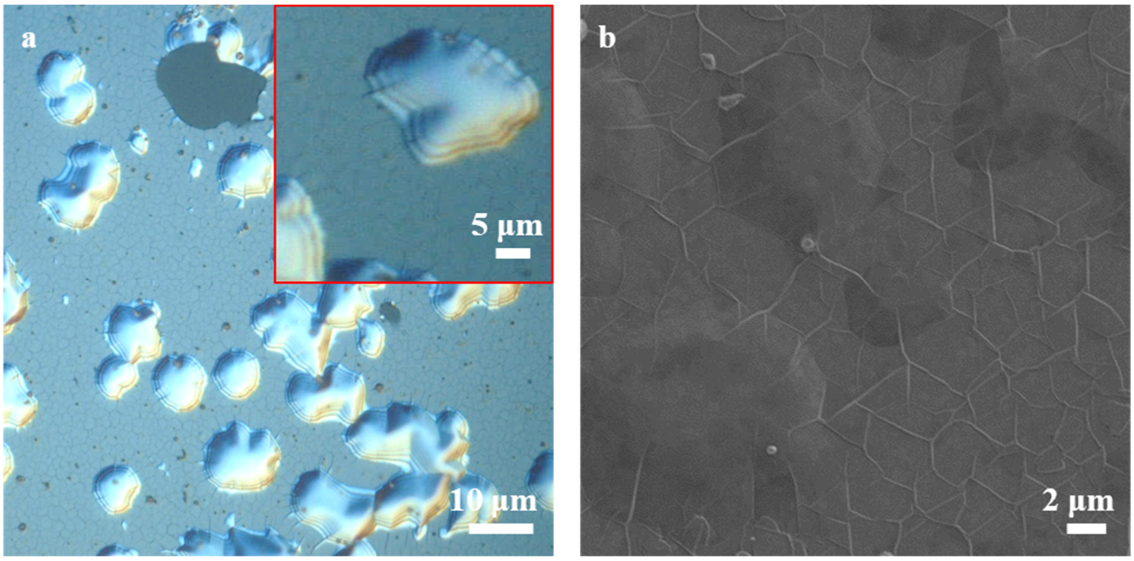


**Supplementary Figure 4 | Characterization of multilayer *h*-BN bubbles. a,** OM image of typical *h*-BN bubbles formed in the growth process. In the inset, the zoom-in image shows clear newton rings. **b,** Corresponding SEM image of the bubble surface.

## Multilayered *h*-BN grown on different substrates

In order to evaluate the generality of this method, multilayer *h*-BN were synthesized on both sapphire and quartz. Corresponding SEM images were shown in supplementary Fig. 5a and b. Because the in-plane thermal expansion coefficient of *h*-BN is $-2.7\times10^{-6}$ $K^{-1}$ [3] while the coefficient of sapphire is positive ($5\times10^{-6}$ $K^{-1}$) [4], the huge difference in thermal coefficient could cause a compressive strain in *h*-BN during cooling after growth, and finally honeycomb wrinkles forms in *h*-BN films obtained. Meanwhile, *h*-BN films synthesized on quartz substrate shows lower density of hexagonal wrinkle patterns, due to the low expansion coefficient of quartz ($0.55\times10^{-6}$ $K^{-1}$) (supplementary Fig. 5e) [5]. Multilayer *h*-BN films on both substrates are in absence of any wrinkled structures after transferred to $SiO_2$ (300 nm)/Si substrate (supplementary Fig. 5c and d). Corresponding Raman spectra of the $E_{2g}$ peak also shows similar features in two samples, which further confirms the generality of this synthesis method (supplementary Fig. 5f).

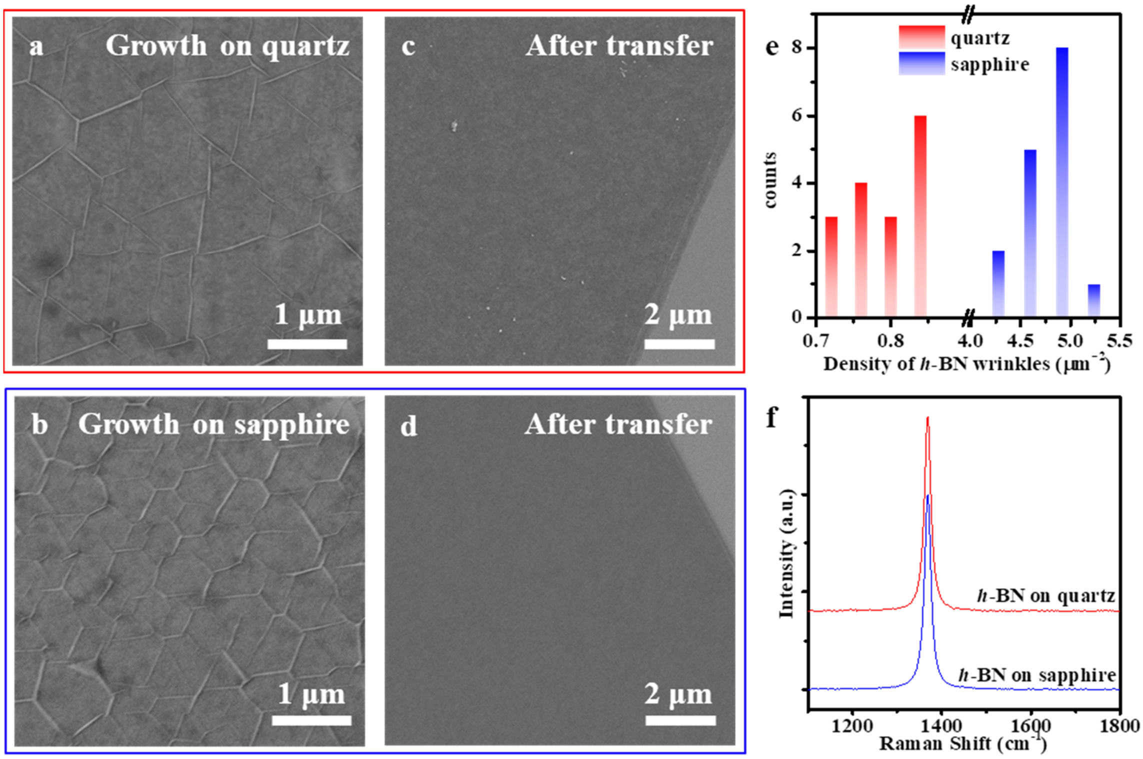


**Supplementary Figure 5 | Multilayer *h*-BN grown on sapphire and quartz. a and b,** Typical SEM images of multilayer *h*-BN grown on quartz (**a**) and sapphire (**b**), respectively. The growth temperature and growth time are fixed at 1250 ºC and 30 minutes in both experiments. **c and d,** Typical SEM of multilayer *h*-BN transferred onto $SiO_2$ (300 nm)/Si substrate corresponding to (**a and b**). **e,** Histogram of the density of *h*-BN wrinkles on quartz and sapphire. The statistical data are obtained randomly from 16 areas of 25 $\mu m^2$ on each substrate. **f,** Typical Raman spectra of multilayer *h*-BN grown on quartz and sapphire, respectively.

### Determination of the uniformity of as-transferred multilayered *h*-BN

We employed AFM to investigate the thickness distribution, which gives the information of the uniformity of *h*-BN films. As shown in Supplementary Fig. 6, two pieces of *h*-BN film in the thickness of 7.1 nm and 39.6 nm are examined by AFM, respectively. Line profiles are randomly extracted and plotted below the corresponding AFM image. The results indicate that the *h*-BN film is uniformity in the thickness.

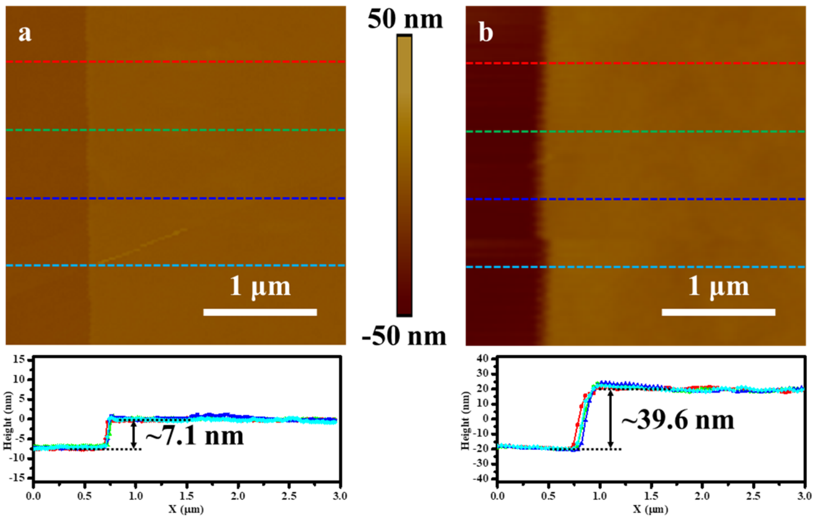


Supplementary Figure 6 | **Thickness uniformity of multilayer *h*-BN.** AFM images of multilayer *h*-BN in the thickness of 7.1 nm **(a)** and 39.6 nm **(b)**, respectively. Line profiles are given below the AFM images.

We also investigated the thickness of as-transferred *h*-BN thin film on top of $SiO_2$ (300 nm)/ Si substrate. Homogenous optical contrast of the film shows the relative high uniformity in thickness of *h*-BN films (Supplementary Fig. 7a). After that, we randomly selected 5 different locations to measure the surface roughness by AFM in tapping mode. As shown in Supplementary Fig. 7b-3f, the AFM images in each 10*10 $\mu m^2$ area shows highly uniformity and the root-mean-square (RMS) demonstrates an ultra-flat surface. Corresponding line profile also indicates an atomic level fluctuation (Supplementary Fig. 7g). Besides, histograms of the height distribution measured by AFM reveal that the as-transferred *h*-BN film has a highly smooth surface (Supplementary Fig. 7h), similar to that of bare sapphire substrates. We added this part into the supplementary information.

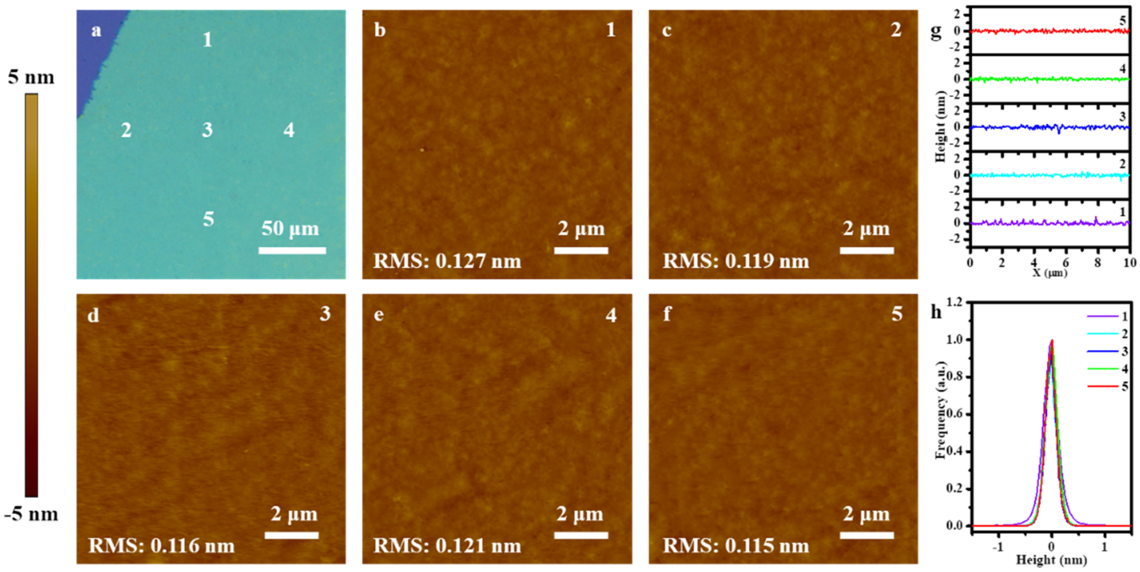


Supplementary Figure 7 | **Surface roughness and uniformity of multilayer *h*-BN. a**, OM image of multilayer *h*-BN on $SiO_2$ (300 nm)/Si substrate. **b-f**, AFM images of multilayer *h*-BN corresponding to **a**. **g-h**, Line profile (**g**) and height distribution (**h**) of AFM images corresponding to **b-f**.

We further investigated the surface roughness of *h*-BN thin films grown under different conditions with AFM. As shown in Supplementary Fig.8a-c, multilayer *h*-BN was synthesized for 60 minutes at different temperatures. The surface roughness of the *h*-BN thin films exhibits atomically level fluctuation, which is comparable with sapphire substrate. The histogram of the height distribution also reveals the as-transferred *h*-BN thin films have highly smooth surface (Supplementary Fig.8d). Besides, the higher temperature always yields higher growth rate, which may yield some three-dimensional *h*-BN accumulations. Thus, the RMS of multilayer *h*-BN would increase slightly with growth temperature increasing. Further, we also analysis the surface roughness of multilayer *h*-BN grown for different growth time. As shown in Supplementary Fig.8e-g, multilayer *h*-BN films are ultra-smooth. Histogram of the height distribution also shows that there is a very small surface roughness (Supplementary Fig.8h).

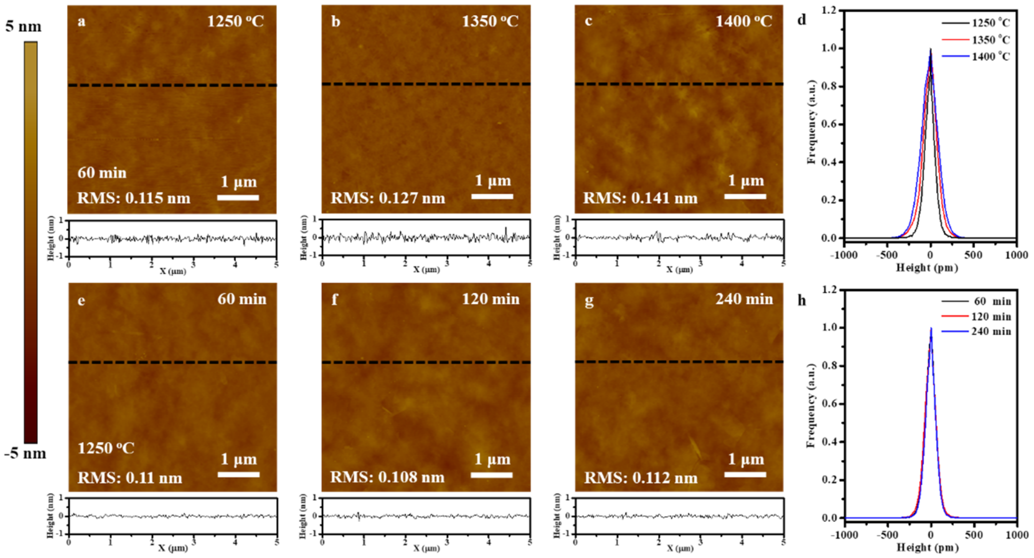


Supplementary Figure 8 | **Investigation on the surface roughness of as-transferred multilayer *h*-BN. a-c**, AFM images of as-transferred multilayer *h*-BN on $SiO_2$ (300 nm)/Si substrate. The *h*-BN films were synthesized for 60 minutes at 1250 °C (**a**), 1350 °C (**b**) and 1400 °C (**c**), respectively. **d**, Histogram of the height distribution of as-transferred *h*-BN thin films corresponding to **a-c**. **e-g**, AFM images of as-transferred multilayer *h*-BN on $SiO_2$ (300 nm)/Si substrate. The *h*-BN films were synthesized at 1250 °C for 60 minutes (**e**), 120 minutes (**f**) and 240 minutes (**g**), respectively. **h**, Histogram of the height distribution of *h*-BN thin films shown in **e-g**.

**Multilayer *h*-BN synthesized at different growth temperatures**

As shown in supplementary Fig. 9a-c, the OM images exhibit uniform, and continuous films of *h*-BN on the $SiO_2$ (300 nm)/Si substrates. Corresponding Raman spectra were collected in supplementary Fig. 9d, the intensity of $E_{2g}$ peak raises obviously with the increase of *h*-BN thickness. The morphology and thickness of these samples were determined by optical contrast and AFM measurement. It confirms that the typical thickness of *h*-BN increases with the growth temperature (supplementary Fig. 9e). In addition, as shown in supplementary Fig. 9f, corresponding activity energy was calculated to be 2.46 eV by Arrhenius equation ($k$=A*exp(-$E_a$·$R^{-1}$·$T^{-1}$), $k$ is the growth rate) [6].

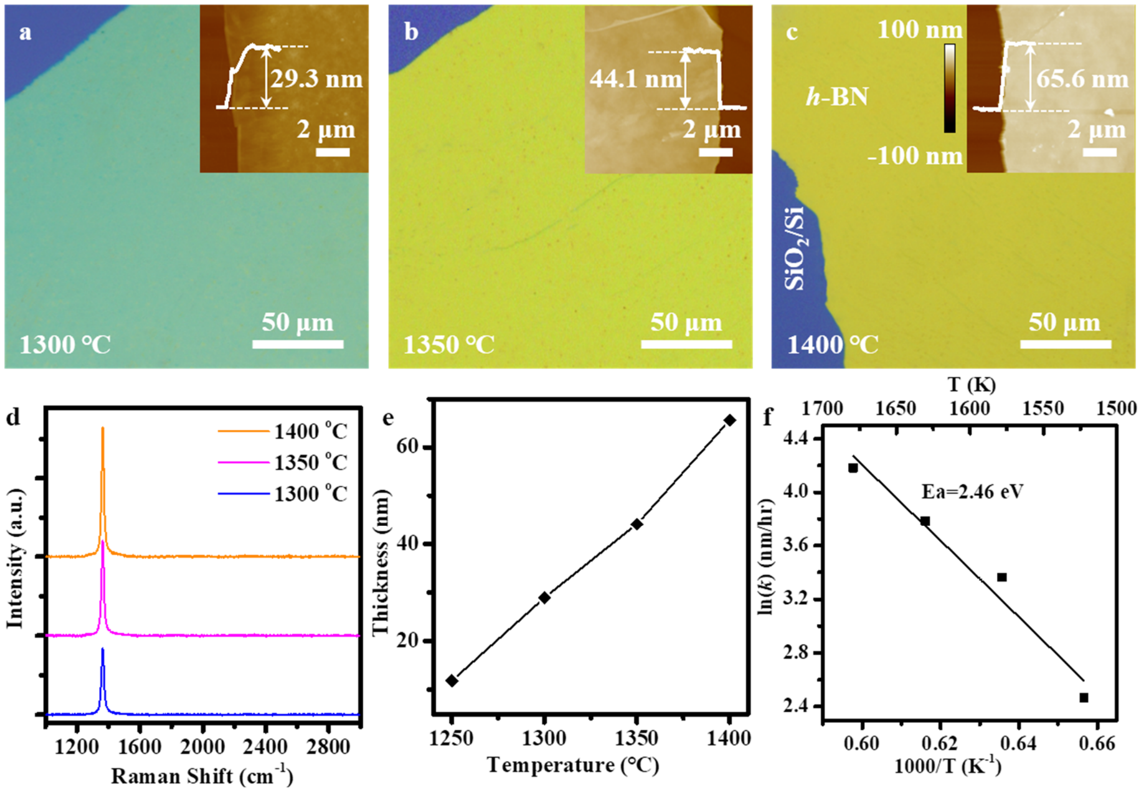


**Supplementary Figure 9 | Multilayer *h*-BN grown on sapphire at different growth temperature. a-c,** OM images of transferred multilayer *h*-BN on $SiO_2$ (300 nm)/Si substrate. Corresponding AFM images determine the heights of multilayer *h*-BN. These multilayer *h*-BN films were obtained at 1300 ºC (**a**), 1350 ºC (**b**) and 1400 ºC (**c**), respectively. **d,** Raman spectra of multilayer *h*-BN, corresponding to (**a-c**). **e,** The relationship between growth temperature and thickness of multilayer *h*-BN. **f,** The relationship between growth temperature and growth rates. According to Arrhenius equation, the activation energy of this reaction is about 2.46 eV.

## Investigation of the phase transition of the Fe-B alloy and *h*-BN growth process

Supplementary Fig. 10a-f show *ex-situ* X-ray photoelectron spectroscopy (XPS) investigation of *h*-BN grown on Fe-B alloy. For multilayer *h*-BN grown on sapphire at 1250 °C, the corresponding XPS spectra of B 1s and N 1s showing the peaks located at 190.0 eV and 397.7 eV, respectively. The calculated B/N atomic ratio is ~1:1, consisted with the multilayer *h*-BN synthesized at 677 °C (950 K). Time-resolved *in-situ* ambient pressure X-ray photoelectron spectroscopy (APXPS) was employed to further investigate *h*-BN growth process during annealing in $N_2/H_2$ atmosphere at 577 °C (850 K). As shown in supplementary Fig. 10g, higher binding energy (BE) and lower BE N peaks at 397.9 eV and 397.5 eV were depicted as the characteristic of monolayer and few-layer *h*-BN, consisting with previous report [7]. With increasing of the reaction time, the intensity of low BE peak increases and the high BE peak decreases gradually. For continuous $N_2$ exposing, the increasing intensity ratio between lower BE peak and higher BE peak indicates that few-layer *h*-BN forms gradually (supplementary Fig. 10h). Briefly, monolayer *h*-BN was evolved into few-layer *h*-BN clearly with increasing of reaction time, which elucidates that isothermal segregation of few-layer *h*-BN phase takes place from B-N associates.

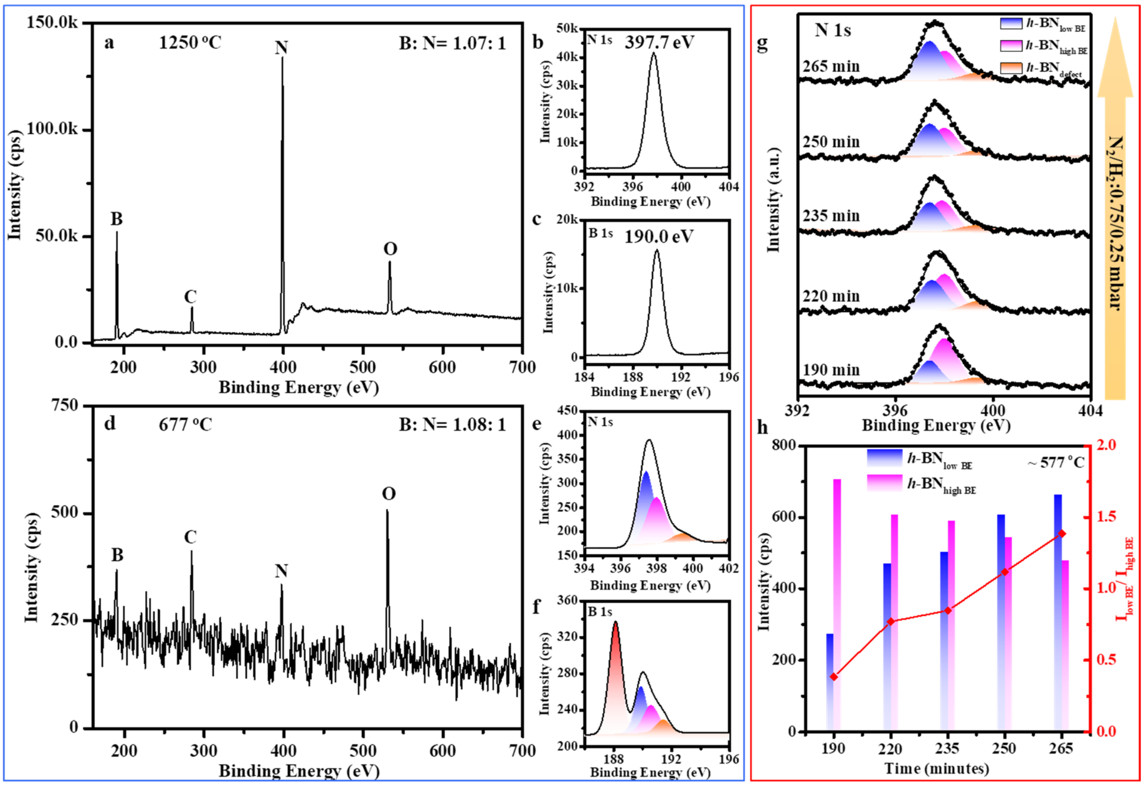


**Supplementary Figure 10 | *Ex-situ* XPS and time-resolved *in-situ* APXPS investigation of *h*-BN grown on Fe-B alloy. a-c,** XPS survey (**a**), N 1s (**b**) and B 1s (**c**) spectra of *h*-BN grown on Fe-B alloy at 1250 °C. **d-f,** XPS survey (**d**), N 1s (**e**) and B 1s (**f**) spectra of *h*-BN grown on Fe-B alloy at 677 °C. **g,** N 1s spectra during the time-

resolved *in-situ* APXPS measurement. **h,** Intensities of lower BE and higher BE N peaks with increasing growth time.

To understand the isothermal growth mechanism, various configurations of cooling conditions including annealed at 1250 ºC, cooled with 10 ºC·min$^{-1}$ and quenched (cooled with around 300 ºC·min$^{-1}$) were carried out to determine isothermal growth process. Typical CVD parameters are used for the *h*-BN growth as shown in Supplementary Fig. 11a, but with three distinctive cooling rates refer to the cooling period from ~1250 ºC down to ~800 ºC. Particularly, for the quenching experiment, the Fe-B alloy and sapphire substrate were sealed into a fused quartz ampoule with 0.02 MPa $N_2$, then the ampoule was loaded into the furnace. After the growth process, the ampoule was dropped out immediately to realize a fast cooling. In these conditions, *h*-BN thin films exhibit similar thicknesses and morphologies (Supplementary Fig. 11b-d). Then, a total of 18 samples transferred onto $SiO_2$ (300 nm)/Si substrate, 6 samples under each experimental condition, was measured through AFM to determine the thickness of *h*-BN films. As shown in Supplementary Fig. 11e, samples annealed at 1250 °C, cooled with 10 °C·min$^{-1}$ and quenched (cooled with around 300 °C·min$^{-1}$) were in the thickness of 27.9 ± 2.5 nm, 26.5 ± 1.9 nm and 26.7 ± 2.2 nm, respectively. It could be observed that total samples exhibit similar thickness distributions. The results indicate that few additional *h*-BN nucleation and multilayers form during cooling, both of which are effects that can be linked to precipitation of B and N species from the bulk [7]. The results may indicate that synthesis of multilayer *h*-BN growth following isothermal growth process.

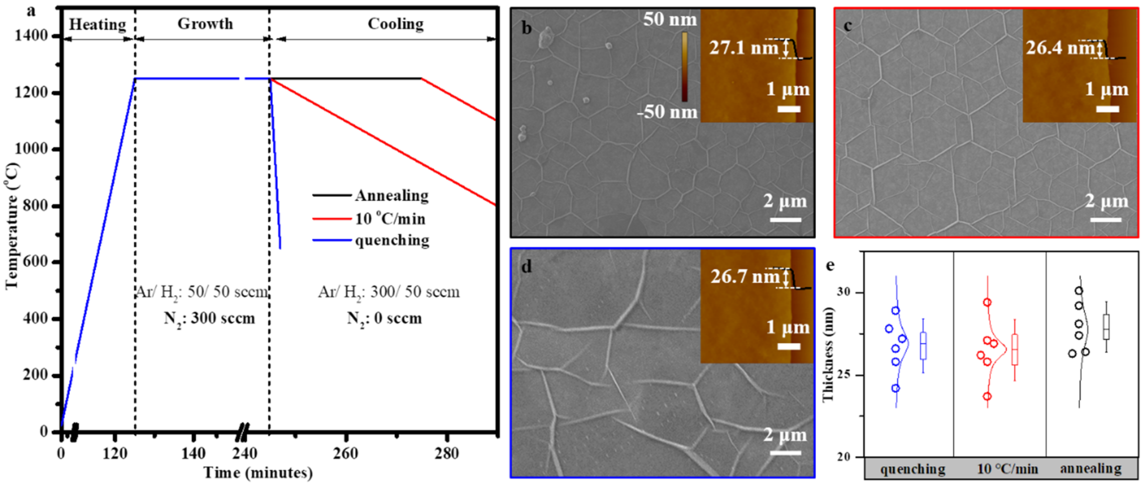


Supplementary Figure 11 | **Influence of cooling rates for synthesizing multilayer *h*-BN. a,** Temperature profile and gas precursor composition with different cooling rates refer to the initial cooling period from ~1250 ºC down to ~800 ºC. **b-d,** SEM images of multilayer *h*-BN on Fe-B alloy with three distinctive cooling rates, which are varied as annealing (**b**), 10 ºC·min$^{-1}$ (**c**) and quenching (**d**), respectively. Insets display corresponding AFM images of as-transferred multilayer *h*-BN on $SiO_2$ (300 nm)/Si substrate. **e,** Thickness distribution of multilayer *h*-BN under different cooling rates.

*In-situ* XRD was employed to understand the phase transition of the Fe-B alloy and *h*-BN growth process during annealing process in $N_2$ atmosphere. As shown in supplementary Fig. 12a, Fe (110) and Fe (200) reflections was observed on the $Fe_{82}B_{18}$ at room temperature. Upon annealing (~900 °C), multiple sharp reflections appear, which can be ascribed to the growth of Fe-borides grains. The formation of $Fe_2B$ is the initial step in the synthesis of *h*-BN products. When the temperature reached to 1100 °C, a peak of *h*-BN at ~26.8° appears with $N_2$ exposure time increasing. It indicates that the isothermal segregation contributes to the *h*-BN growth. The whole process of phase transition was illustrated in supplementary Fig. 12d. After cooling to 900 ºC, the alloy consists of mostly Fe with some Fe-borides phases.

For ternary B-Fe-N system, the isothermal section of ternary B-Fe-N at 900 ºC is presented in supplementary Fig. 12b. BN phase forms from the compound $Fe_2B$ and dissolved nitrogen in Fe-B alloy. According to the phase diagram, as guided by red lines, four-phase equilibrium between Fe, BN, $Fe_2B$, and gas in the temperature is established. In addition, the Fe-B phase transition proved by *in-situ* XRD result is well consistent with the ternary phase diagram (supplementary Fig. 12a). Also, a part of isothermal sections of the Fe corner of the ternary B-Fe-N system at 950 ºC is shown in supplementary Fig. 12c. Major associates to be taken into account in the Fe-rich corner of the liquid phase of the ternary Fe-B-N system are $Fe_2B$ and BN. The results proved that the majority of nitrogen dissolved in the Fe containing B exists in the form of B-N molecules. Only a minor part of the total nitrogen remains atomically dissolved in melts.

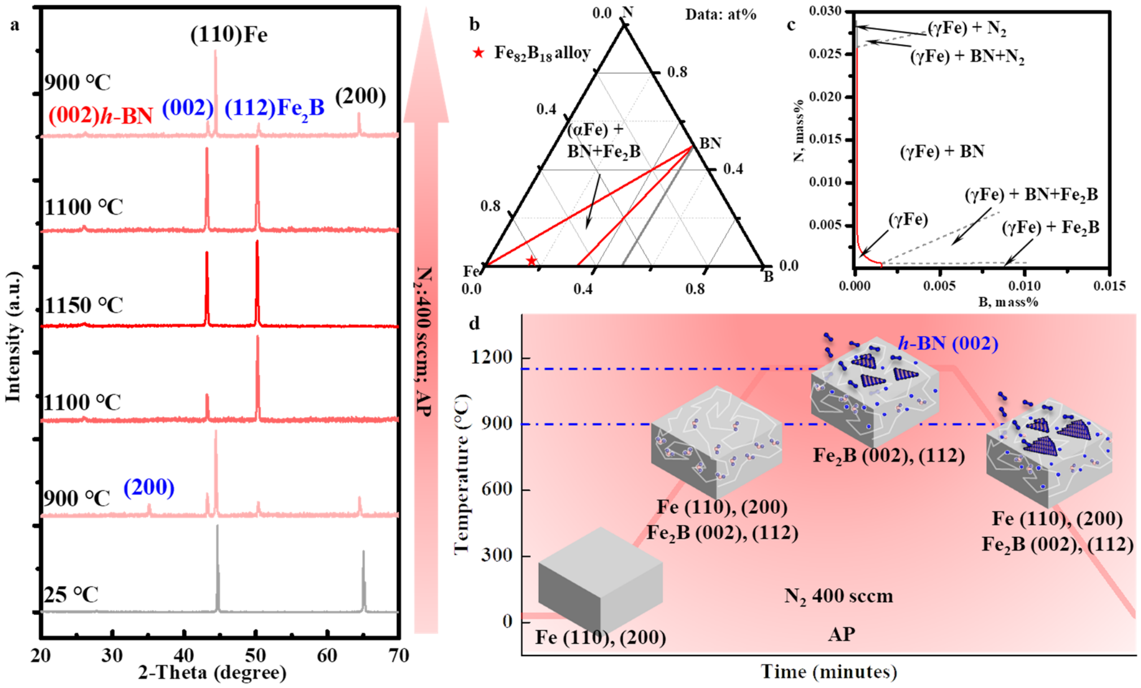

**Supplementary Figure 12 | *In-situ* XRD investigation of *h*-BN grown on Fe-B alloy. a,** XRD spectra of Fe-B alloy during the *in-situ* annealing process. **b,** Isothermal section of B-Fe-N ternary phase diagram at 900 ºC under ambient pressure (adapted from Tomashik, V.) [8]. **c,** The iron corner of the B-Fe-N system at 950 ºC (adapted from Tomashik, V.) [8]. **d,** Schematic of the phase transition during the whole growth process.

Previous literature has reported that a significant amount of nitrogen dissolved in Fe-B melt exists in the form of B-N molecules [8]. In order to understand the nitrogen solubility, the B-Fe-N phase evolution at 1600 ºC is plot in supplementary Fig. 13. As shown in supplementary Fig. 13, the solubility of nitrogen in liquid alloy decreases with increasing boron content. The formation of B-N associates correspondingly in ternary liquid decrease the activity of nitrogen atoms, due to the stabilization of the N in liquid alloy. The activity coefficient of nitrogen ($f_N$) dissolved in infinitely diluted solution of liquid Fe-B alloy is given by:

$$f_N = \left[\frac{\%\text{N (pure Fe)}}{\%\text{N (alloy)}}\right] P_{N_2}, T \qquad (1)$$

The activity coefficient of nitrogen in liquid B-Fe-N alloys increases with increasing of B content in the investigated range 0 to 7 mass% B (see supplementary Fig. 13a). B-N molecules appear and dissolve into the molten surface during the high-temperature annealing [8–10]. The solubility of boron and nitrogen in Fe melts in equilibrium with B-N at 1600 °C and various $N_2$ pressures is shown in supplementary Fig. 13b. The results proved that a significant amount of nitrogen dissolved in Fe-B melt exists in the form of B-N associates. The remaining part of boron (not bonded to nitrogen) is bonded to Fe as $Fe_2B$ and the remaining part of nitrogen (not bonded to boron) is atomically dissolved in melts. Major associates to be considered in the Fe-rich corner of the liquid phase of the ternary Fe-B-N system are $Fe_2B$ and BN.

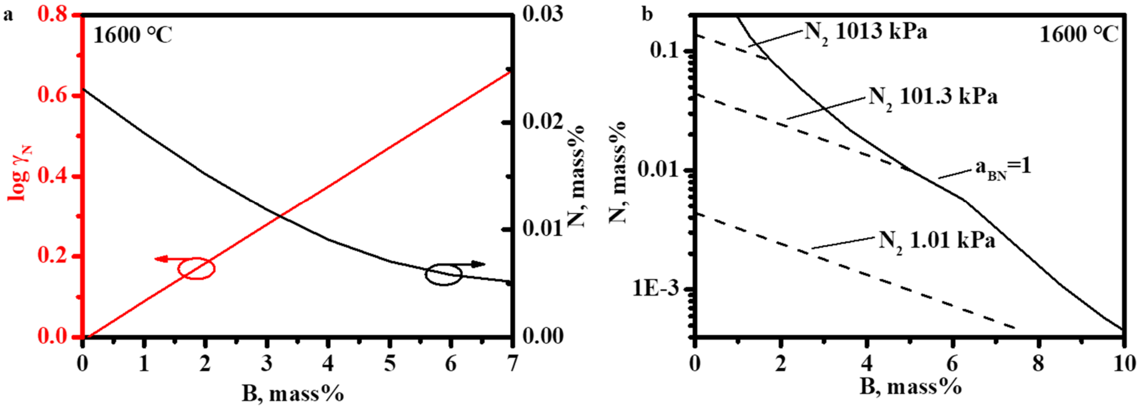


**Supplementary Figure 13 | B-Fe-N phase evolution at 1600 °C. a,** Effect of boron on the nitrogen solubility and nitrogen activity coefficient at 1600 ºC (adapted from Tomashik, V.) [8]. **b,** Equilibria between liquid B-Fe-N alloys and B-N phase at 1600 ºC and various $N_2$ pressure (adapted from Tomashik, V.) [8].

**Investigation of the optically-active defects in multilayer *h*-BN**

In order to determine the distribution of optically-active defects, multilayer *h*-BN and overlapped multilayer *h*-BN thin films were probed by Raman and PL spectroscopy at room temperature. OM, AFM and Raman mapping of $E_{2g}$ mode shows high uniformity of as-synthesized multilayer *h*-BN with assistance of molten $Fe_{82}B_{18}$ alloy (supplementary Fig. 14a and d, and insets in supplementary Fig. 14b and e). PL intensity mapping addresses the defects on *h*-BN flakes (supplementary Fig. 14b and e). Typical PL spectra of multilayer *h*-BN on $SiO_2$ (300 nm)/Si substrate were shown in supplementary Fig. 14c and 14f. The fact that no optical emissions at energies between 1.7 and 2.2 eV could be found in large uniform areas provides the possibility to artificially fabricate optically active defects for optoelectronics in the future.

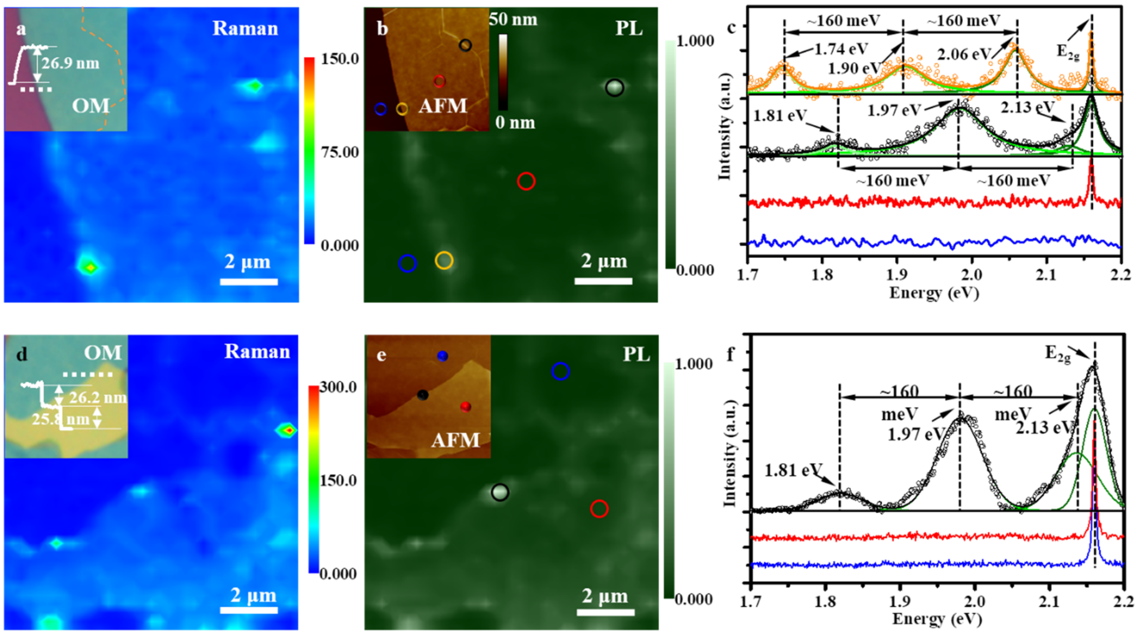


**Supplementary Figure 14 | Investigation of the optically active defects in multilayer *h*-BN. a,b,** Typical Raman mapping of $E_{2g}$ and PL mapping (integrating from 1.7 eV to 2.2 eV) of multilayer *h*-BN on $SiO_2$ (300 nm)/Si substrate. Corresponding OM and AFM images are presented in the insets. **c**, Corresponding PL spectra are extracted from the spots selected in (**b**). **d,** Raman mapping of $E_{2g}$ mode of overlapped multilayer *h*-BN, the inset shows corresponding OM image. **e,** PL intensity mapping addresses the defects on multilayer *h*-BN, the inset shows corresponding AFM image. **f,** PL spectra taken from the positions selected in (e).

**Fabrication of *h*-BN/Graphene/*h*-BN hetero-structure**

For applying the top *h*-BN, firstly, *h*-BN/sapphire is coated with ~1 μm of poly-propylene carbonate (PPC). Then, PPC/*h*-BN is manually peeled and placed onto a transparent PDMS (poly dimethyl siloxane) stamp. Further, the PDMS/PPC/*h*-BN layer was inverted and employed to pick up graphene selected.

For constructing the bottom *h*-BN, similarly, the *h*-BN/sapphire is coated with ~80 nm of poly methyl methacrylate (PMMA, 950 A2), Secondly, PMMA/*h*-BN is manually peeled using Nitto tape. After that, blue tape/PMMA/*h*-BN was placed on $SiO_2$ (300 nm)/Si substrate. Then, hot acetone (80 °C) and thermal annealing (400 °C, Ar/$H_2$ atmosphere) are used to remove blue tape, PMMA and residue contaminations.

PDMS/PPC/*h*-BN/Graphene is placed onto *h*-BN (bottom)/$SiO_2$ (300 nm)/Si substrate precisely through home-made micromanipulator. Finally, the substrate is heated to 80 °C to remove PDMS and then rinsed in acetone to remove PPC, the hetero-structure is left on the substrate.

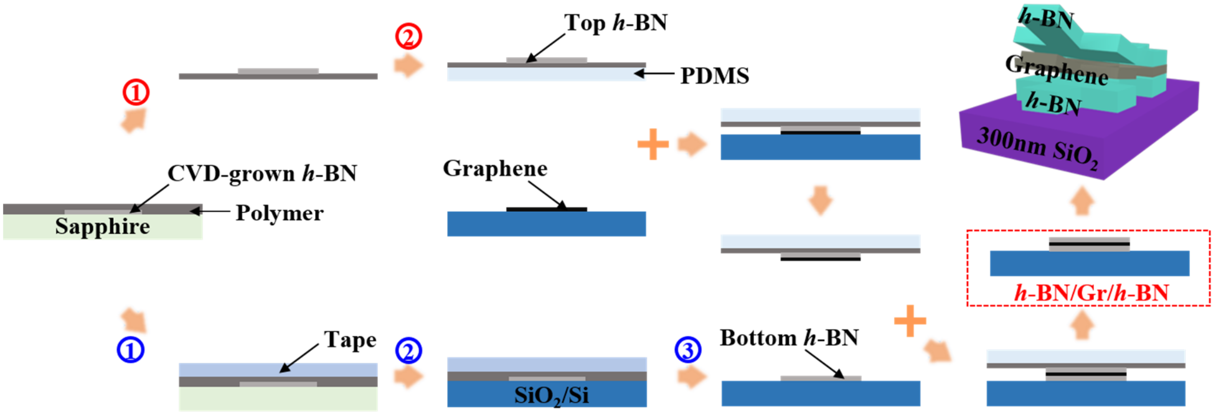


**Supplementary Figure 15 | Fabrication procedure of *h*-BN/Graphene/*h*-BN hetero-structure.**

**Investigation of the carrier mobility of CVD-grown graphene on a $SiO_2$ (300nm)/Si substrate**

To investigate the carrier mobility of CVD-grown graphene without *h*-BN encapsulating, nanofabrication processes were carried out to define the electronic device with metallic contact of Ti (10 nm)/Au (100 nm), and the channel width is 1 μm and the length is about 3 μm. The fabrication processes are the same with that for encapsulated *h*-BN/graphene/*h*-BN.

As shown in supplementary Fig. 16a, the resistance of a typical device as a function of back-gate voltage ($V_{gate}$) was measured at 27 ºC (300 K) under a magnetic field of 9 T. The charge neutrality point located at about 25 V indicates that the graphene channel was p-type doped, which may be induced by trapping charges in the substrate or the residues below graphene. As shown in supplementary Fig. 16b, inset displays an optical image of a graphene device with Hall bar configuration. The mobility of graphene device is ~0.32 to ~0.41 $m^2 \cdot V^{-1} \cdot s^{-1}$ on $SiO_2$ (300 nm)/Si substrate for both holes and electrons.

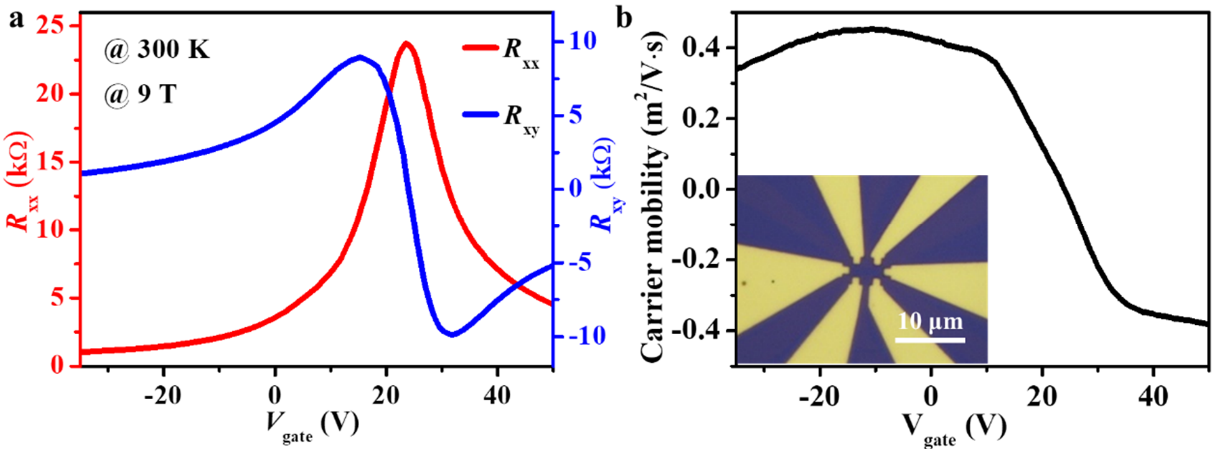


**Supplementary Figure 16 | Electrical properties of CVD-grown graphene transferred on a Si substrate with 300 nm $SiO_2$. a**, The transfer characteristics of the graphene field effect transistor device. **b**, The carrier mobility versus $V_{gate}$. Inset shows the optical image of a typical device.

**The effects of different synthesis conditions on the growth of multilayer *h*-BN**

Here, we systematically investigate the influences of growth conditions on thickness and crystalline quality of the wrinkled *h*-BN on sapphire substrate. It should be pointed that the FWHM of $E_{2g}$ peak were collected from the as-transferred *h*-BN on $SiO_2$ (300 nm)/Si substrate. Some data are listed in Supplementary Table 1. It is obvious that the thickness of *h*-BN films is controllable by adjusting the growth time and temperature.

| Experiments | Temperature (ºC) | Growth time (minutes) | Thickness (nm) | Growth rate (nm/hour) | FWHM of $E_{2g}$ peak ($cm^{-1}$) | FWHM of (0002) peak (°) |
|---|---|---|---|---|---|---|
| #1 | 1250 | 60 | 10.2 | 10.2 | 14.1 | 0.37 |
| #2 | 1250 | 120 | 23.7 | 11.8 | 13.5 | 0.35 |
| #3 | 1250 | 180 | 34.3 | 11.4 | 12.0 | 0.26 |
| #4 | 1250 | 240 | 46.1 | 11.5 | 10.4 | 0.18 |
| #5 | 1300 | 60 | 29.3 | 29.3 | 12.4 | 0.29 |
| #6 | 1350 | 60 | 44.1 | 44.1 | 11.8 | 0.22 |
| #7 | 1400 | 60 | 65.6 | 65.6 | 12.1 | 0.21 |

**Supplementary Table 1 | Summary of multilayer *h*-BN growth under different growth conditions**

## Reference


1. Kobayashi, Y., Tsai, C.-L. & Akasaka, T. Optical Band Gap of *h*-BN Epitaxial Film Grown on ***c***-Plane Sapphire Substrate. *Phys. status solidi* **7,** 1906–1908 (2010).
2. Kumbhakar, P. *et al.* Nonlinear Optical Properties and Temperature-Dependent UV-Vis Absorption and Photoluminescence Emission in 2D Hexagonal Boron Nitride Nanosheets. *Adv. Opt. Mater.* **3,** 828–835 (2015).
3. Tay, R. Y. *et al.* Direct Growth of Nanocrystalline Hexagonal Boron Nitride Films on Dielectric Substrates. *Appl. Phys. Lett.* **106,** 101901 (2015).
4. Paszkowicz, W., Pelka, J. B., Knapp, M., Szyszko, T. & Podsiadlo, S. Lattice Parameters and Anisotropic Thermal Expansion of Hexagonal Boron Nitride in the 10-297.5 K Temperature Range. *Appl. Phys. A Mater. Sci. Process.* **75,** 431–435 (2002).
5. Liu, L. & Edgar, J. H. Substrates for Gallium Nitride Epitaxy. *Mater. Sci. Eng. R Reports* **37,** 61–127 (2002).

6. Retajczyk, T. F. & Sinha, A. K. Elastic Stiffness and Thermal Expansion Coefficients of Various Refractory Silicides and Silicon Nitride Films. *Thin Solid Films* **70,** 241–247 (1980).
7. Caneva, S. *et al.* Controlling Catalyst Bulk Reservoir Effects for Monolayer Hexagonal Boron Nitride CVD. *Nano Lett.* **16,** 1250–1261 (2016).
8. Tomashik, V. in (eds. Effenberg, G. & Ilyenko, S.) **11D1,** 457–471 (Springer Berlin Heidelberg, 2008).
9. Evans, D. B. & Pehlke, R. D. The Boron-Nitrogen Equilibrium in Liquid Iron. *Transactions of the Metallurgical Society of AIME*. **230**, 1657-1662 (1963).
10. Fountain. Solubilty and Precipitation of Boron Nitride in Iron Boron Alloys. *Transactions of the Metallurgical Society of AIME.* **243,** (1962).
11. Castellanos-Gomez, A. *et al.* Local Strain Engineering in Atomically Thin $MoS_2$. *Nano Lett.* **13,** 5361–5366 (2013).
12. Li, H. *et al.* Optoelectronic Crystal of Artificial Atoms in Strain-Textured Molybdenum Disulphide. *Nat. Commun.* **6,** (2015).
